# Epistatic Effect of Regulators to the Adaptive Growth of *Escherichia coli*

**DOI:** 10.1038/s41598-020-60353-3

**Published:** 2020-02-27

**Authors:** Yukari Miyake, Kaneyoshi Yamamoto

**Affiliations:** 1 Hosei University, Department of Frontier Bioscience, Koganei, Tokyo, 184-8584 Japan; 2 Hosei University, Research Institute of Micro-Nano Technology, Koganei, Tokyo, 184-8584 Japan

**Keywords:** Genetic engineering, Synthetic biology

## Abstract

Bacteria survive in the environment with three steps: a sensing environmental conditions, a responding to sensed signals, and an adaptation for proper survival in the environment. An adapting bacterial cell occurs cell division to increase the number of sister cells, termed adaptive growth. Two-component systems (TCSs), representing the main bacterial signal transduction systems, consist of a pair of one sensor kinase (SK) and one response regulator (RR), and RR genes are abundant in most bacterial genomes as part of the core genome. The OmpR gene family, a group of RR genes, is conserved in 95% of known bacterial genomes. The *Escherichia coli* genome has an estimated 34 RR genes in total, including 14 genes of OmpR family genes. To reveal the contribution of TCSs for fast growth as an adaptive growth strategy of *E. coli*, we isolated a set of gene knockout strains by using newly developed genome editing technology, the HoSeI (Homologous Sequence Integration) method, based on CRISPR-Cas9. The statistics of single cell observation show a knockout of an arbitrary pair of *phoP, phoB*, and *ompR* genes, stably expressed by positive feedback regulation, dramatically inhibit the optimum adaptive growth of *E. coli*. These insights suggest that the adaptive growth of bacteria is fulfilled by the optimum high intracellular level of regulators acquired during growth under environmental conditions.

## Introduction

Bacteria survive and then increase their population by binary cell division in a timely manner. Individual bacterial cells sense changing environmental signals, transduce those environmental signals into biological responses, and adapt to the environmental change by biological responses. Successful integration of these biological stress responses must induce adaptive growth to obtain a continuous chance for the production of offspring. Bacteria are able to survive in various environments by changing the expression pattern of their genome, which takes place by controlling the promoter recognition properties of RNA polymerase by transcription factors^1^. Transcription factors (TFs) are classified as those that facilitate direct sensing and indirect sensing of environmental signals. The Lac repressor and Crp activator are good examples of direct sensing TFs and sense lactose and cAMP, respectively. A response regulator (RR) is a typical example of an indirect sensing TF and is activated by phosphorylation by the cognate sensor kinase (SK), detecting environmental signals at the membrane^1,2^. Direct sensing TFs temporally support bacterial growth because extracellular nutrients stimulate intracellular metabolism and produce signal metabolites that activate those TFs.Indirect sensing TFs, such as RR, are known to be important for temporal survival but are not known for aiding continuous growth.

Recent massive amounts of genomic information show the core genome, a set of species-specific genes, and pan genome, representing non-conserved genes^3^. Within the core genome, there is a set of genes involved in central and secondary metabolism, cell cycle, and gene expression, many of which are essential for growth^3^. In addition to genes coding for essential biological functions, regulatory genes are conserved in the core genome but are not essential for growth^3^. One of the core-genome regulatory genes is the RR gene, which is a unique signal transduction component that is only conserved among prokaryotes. Two-component systems, known as the common signal transduction systems of prokaryotes, basically consist of a pair of one SK and one RR. SK autophosphorylates upon stimulus and then transfers a phosphate group to a cognate RR. The typical phosphorylated form of a given RR is active and controls the cellular system to survive in changing environments by gene expression regulation because most response regulators harbour structural domains for DNA binding to regulate genome expression^4^. Several TFs are autoregulated during the response to environmental signals. In *Escherichia coli*, more than 50% of identified TFs in the genome are positively or negatively autoregulated^5^. Positive autoregulation leads to a fast response and impacts the response dynamics^6,7^. Among ~30 positively autoregulated TFs in the *E. coli* genome^5^, 10 of those are RRs of TCSs, and 34 RRs have been characterized in the *E. coli* genome. On the other hand, negatively autoregulated RRs are less common than positively autoregulated RRs in TCSs^8^. A recent report showed that coupled positive and negative feedback allowed both a fast response and optimal RR protein levels in the PhoB/PhoR system in *E. coli*^6^. However, the contribution of coupled autoregulation for continuous bacterial growth is poorly understood.

We found a correlation of the RR family with bacterial genome size, speculating that the main RR genes (*phoP, phoB, ompR*) affect *E. coli* growth. To gain insight into the effect of RR genes on growth, we isolated the set of all combinations of single-, double-, and triple-gene knockout strains by using the newly developed HoSeI method for the *E. coli* genome based on CRISPR-Cas (clustered regularly interspaced short palindromic repeats CRISPR-associated proteins 9). The differences of elapsed time until the first cell division and initial growth rate of the isolated strains were measured and analysed with statistics, indicating that a particular pair of RR genes of *E. coli* K-12 are required for adaptive growth via genetic epistasis.

## Results

### Estimation of highly conserved response regulator genes in the bacterial genome

**Figure 1:**
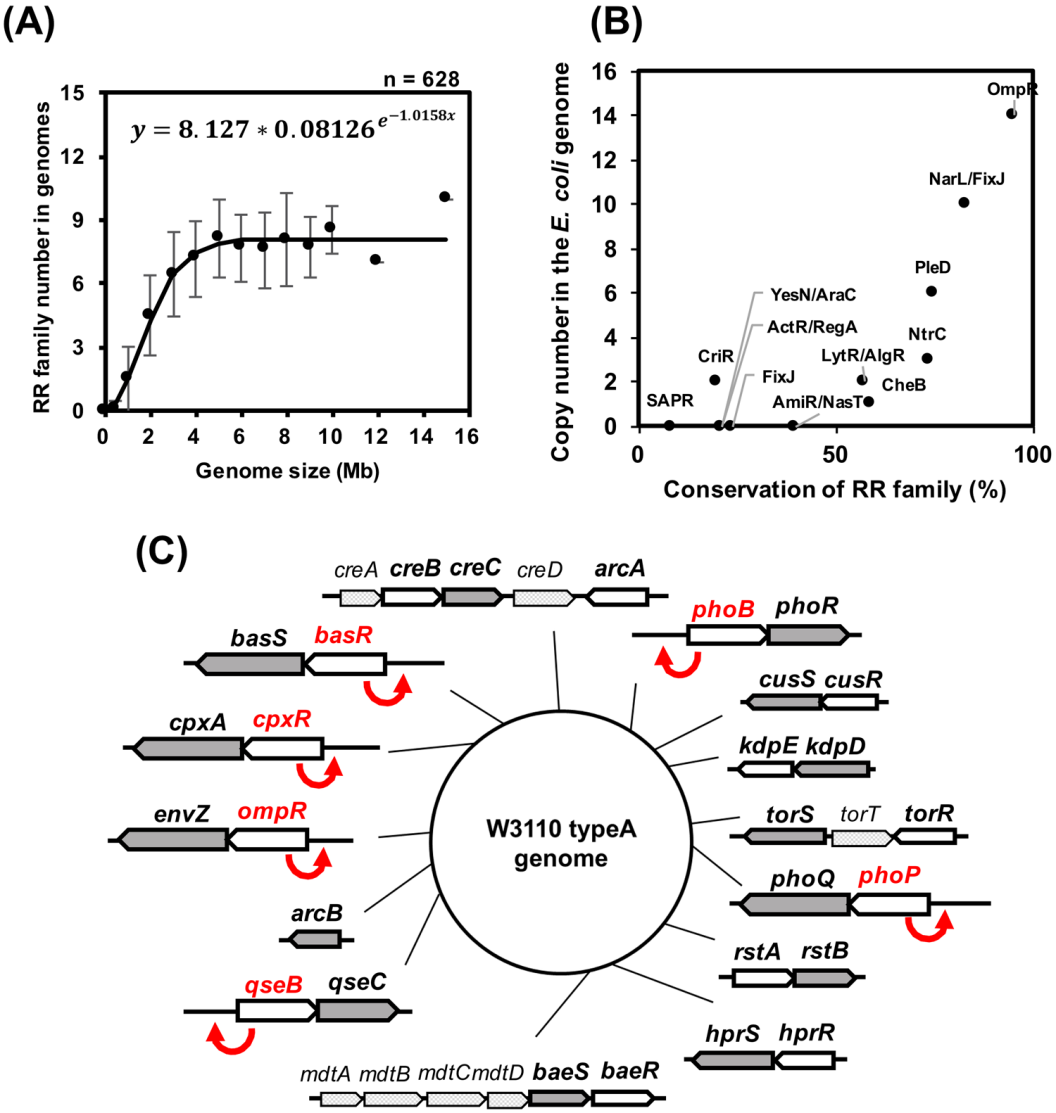
Conservation of two-component system response regulator families in bacterial genomes. (**A**) The correlation between genome size and the number of RR families in the genomes. Twelve groups are detected as two-component response regulators from the bacterial genomes of 628 species in the COG database as follows: OmpR family as COG0745, NarL/FixJ family as COG2197, PleD family as COG3706, NtrC family as COG2204, CheB family as COG2201, LytR/AlgR family as COG3279, AmiR/NasT family as COG3707, FixJ family as COG4566, ActR/RegA family as COG4567, YesN/AraC family as COG4753, CriR family as COG4565, and SAPR family as COG3947. The number of COGs involved in the RR family (y-axis) was analysed in comparison with the genome size (x-axis). The average number of COGs (black circle) and standard deviation (SD, error bar) were calculated for each genome size of 0.5, 1, 2, 3, 4, 5, 6, 7, 8, 9, 10, 12, and 15 Mb. The calculated data were fitted by the Gompertz curve $y=a{b}^{{e}^{-cx}}$, where y is the number of COGs and x is the genome size. The formula of the fitted Gompertz curve is shown in the graph. (**B**) Conservation of RR families in the bacterial genomes and gene copy number in the *E. coli* genome. Based on the calculated data shown in (**A**), the conservation (%) of each COG was calculated on all genomes of 628 species of bacteria (x-axis). The y-axis shows the gene copy number of each RR family in the *E. coli* K-12 genome. (**C**) OmpR family RR genes in the *E. coli* K-12 genome. *E. coli* K-12 W3110 has 14 OmpR family response regulator genes in its genome. Each arrow shows OmpR family response regulators (white), their cognate sensor kinases (gray), and other genes in the operon (light gray). The direction of arrows shows the direction of genes in the *E. coli* genome. Red letters and arrows show the genes known as positive feedback regulators.

The Clusters of Orthologous Groups of proteins (COGs) database has been designed to classify proteins on the basis of orthology^9^. Twelve groups are detected as two-component response regulators from the genomes of 628 bacteria species in the COG database^10^ as follows: OmpR family as COG0745, NarL/FixJ family as COG2197, PleD family as COG3706, NtrC family as COG2204, CheB family as COG2201, LytR/AlgR family as COG3279, AmiR/NasT family as COG3707, FixJ family as COG4566, ActR/RegA family as COG4567, YesN/AraC family as COG4753, CriR family as COG4565, and SAPR family as COG3947. We found a correlation between the number of RR COGs and genome size, as fitted by the Gompertz curve (Fig. 1A). Bacterial genomes less than 5 Mbp in size contained the relative RR COG number of 2 RR COGs per 1 Mbp of genome, while bacterial genomes more than 5 Mbp in size contained 8 RR COGs (Fig. 1A). Bacterial RNA polymerase core enzyme consist of 2*α*, *β*, *β*′, *ω*^11^, which were featured by COG0202, COG0085, COG0086, and COG1758^10^. The COG0202, COG0085, and COG0086 were almost completely conserved among more than 99% of bacteria registered in COG database^10^. In addition of four COGs featuring bacterial RNA polymerase core subunits, the 8 COGs of RNA polymerase subunits were similarly analysed, resulting that bacterial genomes contained at least 4 RNA polymerase COGs relating RNA polymerase core subunits and major sigma factor and bacterial genomes more than 3 Mbp in size contained 7 RNA polymerase COGs by the addition of COGs relating minor sigma factors (Fig. S1). Among the 628 species of bacteria, conservation of RR COGs is the highest in the OmpR family, at 95%, and the lowest in the SAPR family, at 8.4% (Fig. 1B), suggesting that the OmpR family is extensively conserved in bacteria as a member of the core genome.

The copy number of RR genes of the *E. coli* K-12 genome was evaluated in detail. The genome sequence of *E. coli* K-12, which is 4.6 Mbp in size, has 34 predicted response regulator genes, of which 6 are classified as RR COGs^12,13^. Among all 34 RR genes, OmpR family genes are the most prevalent, with 14 genes, *arcA, phoB, cusR, kdpE, torR, phoP, rstA, hprR, basR, qseB, ompR, cpxR, creB*, and *baeR*, and the CriR family and LytT family have the smallest number, at 2 genes, with* citB* and *dcuR* and with *btsR* and *pyrR*, respectively (Figs. 1B and S2A). Six RR genes of the OmpR family are known to be positively self-activated (Fig. 1C)^14–19^. In good agreement with the positive feedback regulation of the OmpR family RRs of *E. coli* K-12, OmpR, PhoB, and PhoP are abundant regulators in *E. coli* through the growth phase in rich and poor medium, with approximately 1,000 copies per genome^20^ (Fig. S2B). Taking these observations together, we suspected that OmpR, PhoB, and PhoP play important roles in the adaptive growth of fast-growing *E. coli* as main RR factors.

### Homologous sequence integration (HoSeI) method for multi-gene knockout in the *E. coli* genome by introduction of a nonsense codon using CRISPR-Cas

The CRISPR-Cas system is available to knock out genes in the *E. coli* genome by insertion of an antibiotic resistance marker gene^21^. We developed a novel gene knockout system by introducing a nonsense codon, a homologous sequence integration (HoSeI) method, to overcome the limitations of antibiotic resistance marker genes. It is readily feasible for us to design and isolate multi-gene knockout strains of *E. coli*. The designed sgRNA (single-guide RNA) containing the recognition sequence for the *E. coli* genome specifically digests the genome, resulting in a dead phenotype (Fig. S3A). The designed DNA fragment containing the following sequence, however, recombines with the genome at the injury site by lambda-Red recombinase, resulting in a living phenotype (Fig. S3A). To knock out gene function, the DNA fragment is designed to introduce the stop codon, TAA, instead of the PAM sequence (Fig. S3B). For knockout of each gene, we constructed sgRNA-expressing plasmids (Table S2). None of the constructed psgRNA plasmids caused *E. coli* harbouring pCas to gain ampicillin resistance (data not shown). The addition of the designed DNA fragment, including the nonsense codon for the target gene, recovered the transformation of *E. coli* harbouring pCas by psgRNA. The proper introduction of the nonsense codon, TAA, was confirmed by Sanger sequencing in all cases (Fig. S4). We isolated a psgRNA-free transformant by IPTG-inducible sgRNA for the psgRNA plasmid from pCas as previously described^21^. The isolated psgRNA-free transformant was subjected to further gene knockout by repeating the HoSeI method. In addition to all single knockouts of the 14 OmpR family RRs, these experiments bred all strains of seven combinations of main RR gene knockout for PhoB, PhoP, and OmpR (Table S1).

### Contribution of the optimum adaptive growth of *E. coli* by RR genes

**Figure 2:**
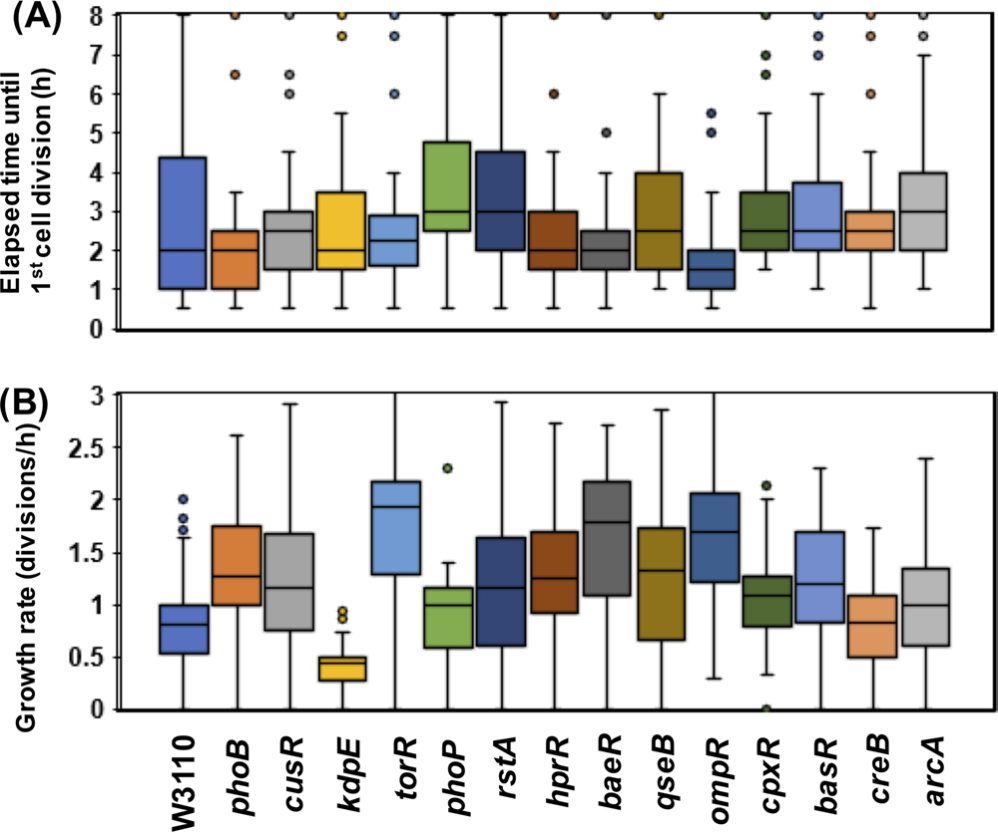
Contribution of OmpR family RRs to the adaptive growth of *E. coli* cells. Time-lapse observation of single cells growing on solid medium was adopted in the 30 min range for 8 hours with a light microscope (Fig. S5). Based on the measured data (Fig. S5), the elapsed time until cell division (hours, shown in **A**) and growth rate of single *E. coli* cells (divisions/hour, shown in **B**) were calculated and are shown as box plots with error bars and outliers.

Bacteria are able to grow both in liquid and solid conditions. In both cases, the number of bacteria cells similarly increases^22^. To measure the elapsed time until cell division and growth rate of single *E. coli* cells, time-lapse observation of single cells growing on solid medium was adopted in a 30 min range for 8 h with light microscopy. At the end of observation after 8 hours, 70.8% of the parent strain, W3110 typeA, cells formed micro-colonies including 4 to 8 cells, while 8.3% of the cells were not completely divided (Fig. S5a). The majority of parent cells started cell division 0.5–4.5 hours after incubation (80.2%) and showed a growth rate of 0.5–1.1 divisions/hour (75.0%) (Figs. 2 and S5a). Fourteen single OmpR family RR gene knockout strains isolated in this study were subjected to time-lapse observation, as was the parent strain. Most of the single RR gene knockouts, except for Δ *ompR*, showed that the percentage of non-growing cells was in the range of 17% to 1.5% at the end of observation after 8 hours, with a maximum of 17% for Δ *rstA* and a minimum of 1.5% for Δ *cpxR* (Figs. 2A and S5). All of the Δ *ompR* strain cells formed micro-colonies (Figs. 2A and S5k). For all of the single RR gene knockout strains, more than 75% of cells started cell division in the time range of 0.5 - 4.5 hours, similar to the parent strain, with several deviations (Fig. 2A). The growth rates were divided into three groups with comparison to the parent strain: the faster growth rate mutants were Δ *phoB*, Δ *cusR*, Δ *torR*, Δ *rstA*, Δ *hprR*, Δ *baeR*, Δ *qseB*, Δ *ompR*, Δ *cpxR*, Δ *basR*, and Δ *arcA*; the similar growth rate mutants were Δ *phoP* and Δ *creB*; and the slow growth rate mutant was Δ *kdpE* (Fig. 2B).

### The adaptive growth of *E. coli* defected by a knockout of an arbitrary pair of *phoP, phoB*, and *ompR* genes

**Figure 3:**
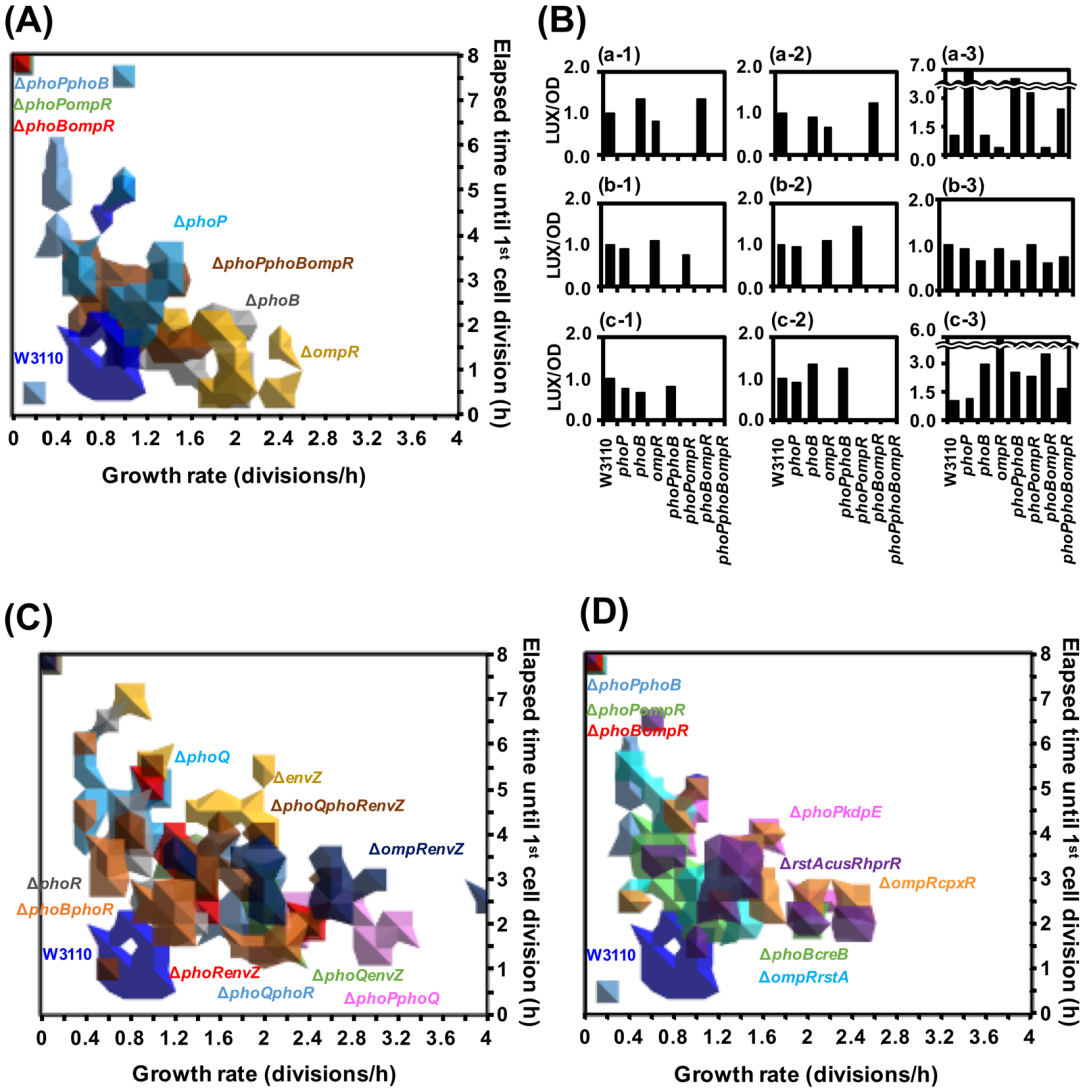
Contribution of main two-component RRs to the adaptive growth of *E. coli* cells. Time-lapse observation of single cells growing on solid medium was adopted in the 30 min range for 8 hours with a light microscope (Fig. S6). Based on the measured data (Fig. S6), the elapsed time until cell division (hours, shown on the y-axis) and growth rate of a single *E. coli* cell (divisions/hour, shown on the x-axis) were calculated and are shown as contour graphs (**A**,**C**, and **D**). (**A**) The parent strain (blue) and three of the main RR (*phoP*, *phoB*, and *ompR*) gene knockout strains, Δ *phoP* (light blue), Δ *phoB* (gray), Δ *ompR* (yellow), Δ *phoP* Δ *phoB* (pale blue), Δ *phoP* Δ *ompR* (yellowish green), Δ *phoB* Δ *ompR* (red), and Δ *phoP* Δ *phoB* Δ *ompR* (dark brown). (**B**) The constructed pLux-mgtA (a), pLux-pstS (b), and pLux-ompC (c), shown in Table S2, were transformed into each strain (a-1,b-1, and c-1) and each strains carrying either RR protein expression plasmids, pBADPhoP-FLAG (a-3), pBADPhoB-FLAG (b-3), or pBADOmpR-FLAG (c-3) or the empty pBAD33 vector (a-2,b-2, and c-2). Transformants were grown in the M9-glucose medium including 50 *μ*g/mL kanamycin for pLUX plamisd and 20 *μ*g/mL chloramphenicol for pBAD plasmid at 37 °C with shaking for overnight. And then, overnight culture was diluted 100-fold by fresh M9-glucose medium with (a-2 and 3, b-2 and 3, and c-2 and 3) and without (a-1,b-1, and c-1) 0.002% arabinose for RR protein expression from pBAD plasmid and incubated at 37 °C with shaking. OD_600_ and a total intensity of luminescence were measured and the ratio of luminescence to OD_600_ (LUX/OD) of each strain was calculated. The specific activity for each promoter was normalized with the parent strain W3110. (**C**) The parent strain (blue), cognate SK genes of main RR (*phoP*, *phoB*, and *ompR*) knockout strains Δ *phoQ* (light blue), Δ *phoR* (gray), Δ *envZ* (yellow), Δ *phoQ* Δ *phoR* (pale blue), Δ *phoQ* Δ *envZ* (yellowish green), Δ *phoR* Δ *envZ* (red), and Δ *phoQ* Δ *phoR* Δ *envZ* (dark brown), and cognate pairs of SK-RR knockout strains Δ *phoP* Δ *phoQ* (pink), Δ *phoB* Δ *phoR* (light brown), and Δ *ompR* Δ *envZ* (navy). (**D**) The parent strain (blue), double RR gene knockout strains Δ *phoP* Δ *phoB* (pale blue), Δ *phoP* Δ *ompR* (yellowish green), Δ *phoB* Δ *ompR* (red), Δ *phoP* Δ *kdpE* (pink), Δ *phoB* Δ *creB* (green), Δ *ompR* Δ *cpxR* (orange), and Δ *ompR* Δ *rstA* (light blue), and a triple RR gene knockout strain Δ *rstA* Δ *cusR* Δ *hprR* (purple).

Next, three double main RR gene knockout strains, Δ *phoP* Δ *phoB*, Δ *phoP* Δ *ompR*, and Δ *phoB* Δ *ompR*, were isolated from two single RR knockout strains, Δ *phoB* and Δ *phoP* (Table S1) and subjected to time-lapse observation to determine the elapsed time until cell division and the growth rate. Most of the cells of all of the three double RR gene knockout strains did not significantly grow at the end of observation after 8 hours (Fig. S6), resulting in a long elapsed time and slow growth rate (Figs. 3A and S6). However, all three double RR gene knockout strains formed colonies after incubation for more than 48 hours under the same conditions (data not shown). Surprisingly, the triple RR gene knockout strain showed similar behaviour to that of the parent strain. At the end of observation after 8 hours, 93.1% of Δ *phoP* Δ *phoB* Δ *ompR* strain cells formed micro-colonies, while 6.9% of them were not totally divided (Fig. S6). The majority of the Δ *phoP* Δ *phoB* Δ *ompR* strain cells started cell division at 1.5–3.5 hours after incubation (86.2%) and showed a growth rate of 0.6–1.4 divisions/hour (79.3%), as did the parent strain (Figs. 3A, S6h and S7).

We examined to confirm gene knockout of an arbitrary pair of *phoP, phoB*, and *ompR* genes, defecting the adaptive growth, by genetic complement test using luciferase reporter system (Fig. 3B). The *ompC*, *pstS*, and *mgtA* promoters were employed as target promoters by EnvZ-OmpR, PhoQ-PhoP, PhoR-PhoB. The activation of *ompC* promoter requires the phosphorylated OmpR to upstream of *ompC* gene, which is stimulated by the cognate SK EnvZ at high osmolarity^1^. The activation of *pstS* and *mgtA* promoters requires the phosphorylated PhoB and PhoP to upstream of each gene, respectively^23,24^. The intracellular level of phosphorylated PhoB is decreased by PhoR at high level of inorganic orthophosphate^23^ and the level of phosphorylated PhoP is decreased by PhoQ at high level of magnesium^24^. Each *lux* reporter plasmid, *ompC-lux*, *pstS-lux*, and *mgtA-lux*, was introduced into all strains of seven combinations of main RR gene knockout for OmpR, PhoB, and PhoP (Table S1) and then the luciferase activity was measured in each culture of M9 glucose. As expected, the activity of *ompC* promoter was detected in the parent strain but not in the knockout strains defecting only *ompR* gene (Fig. 3B). The defect of *ompC* promoter activity was recovered by expression of *ompR* gene in all of the knockout strains defecting only *ompR* gene (Fig. 3B). As well as the activation of *ompC* promoter by *ompR* gene, the activities of *pstS* and *mgtA* promoters were defected in the knockout strains defecting only *phoB* and *phoP* genes, respectively (Fig. 3B). The activities of *pstS* and *mgtA* promoters in mutants were recovered by expression of *phoB* and *phoP* genes, respectively (Fig. 3B).

Taking all these observations together, OmpR, PhoP, and PhoB each slightly contribute to the growth of *E. coli*, while the disappearance of any two OmpR, PhoP, and PhoB proteins seriously disrupts the adaptive growth of fast-growing *E. coli*.

### The adaptive growth of *E. coli* not defected by a knockout of an arbitrary pair of *envZ, phoR*, and *phoQ* genes

EnvZ, PhoR, and PhoQ are the cognate SKs of OmpR, PhoB, and PhoP, respectively. Three single SK gene knockout strains, Δ *envZ*, Δ *phoR*, and Δ *phoQ*, were subjected to time-lapse observation as described above. At the end of observation after 8 hours, the percentages of non-growing cells of Δ *envZ*, Δ *phoR*, and Δ *phoQ* were 32.0%, 20.3%, and 21.5%, respectively (Fig. S6). In the case of Δ *envZ*, 54.0% of cells started cell division 4.0–6.5 hours after incubation, and 50.0% showed a growth rate of 0.8–2.0 divisions/hour, similar to the parent strain (Figs. 3C, S6i and S7). In the case of Δ *phoQ*, 61.5% of cells started cell division 4.0–5.5 hours after incubation, and 67.7% showed a growth rate of 0.3–1.0 divisions/hour, similar to the parent strain (Figs. 3B, S6i and S7). In the case of Δ *phoR*, 62.5% of cells started cell division 1.5–4.5 hours after incubation, and 73.4% showed a growth rate of 0.4–1.4 divisions/hour, similar to the parent strain (Figs. 3C, S6j and S7). Overall, three single SK gene mutants showed slightly delayed elapsed times, but their growth rate was similar to that of the parent strain.

Next, three double SK gene knockout strains, Δ *phoQ* Δ *phoR*, Δ *phoQ* Δ *envZ*, and Δ *phoR* Δ *envZ*, were subjected to time-lapse observation to determine the elapsed time until cell division and the growth rate. Most of the cells of the three double SK gene knockout strains grew significantly at the end of observation after 8 hours (Fig. S6). All three double SK gene knockout strains showed an elapsed time in the range of 2.0–3.5 hours, and the growth rate varied in the range of 0.8–2.4 divisions/hour (Figs. 3B, S6 and S7). In addition, for the triple SK gene knockout strain, 92.6% of cells formed micro-colonies (Fig. S6). The majority of the Δ *phoQ* Δ *phoR* Δ *envZ* strain cells started cell division 1.5–4.5 hours after incubation (77.8%) and showed a growth rate of 0.4–2.0 divisions/hour (74.1%) (Figs. 3C, S6o and S7).

Taking all these observations together, the disappearance of any two OmpR, PhoP, and PhoB proteins seriously disrupts the adaptive growth of fast-growing *E. coli*, while the disappearance of any two EnvZ, PhoR, and PhoQ, the cognate SKs for OmpR, PhoB, and PhoQ, proteins slightly affect the adaptive growth of fast-growing *E. coli*.

### A specific arbitrary pair of *phoP, phoB*, and *ompR* genes for double gene knockout inhibiting the optimum adaptive growth of *E. coli*

All three double RR gene knockout strains, Δ *phoP* Δ *phoB*, Δ *phoP* Δ *ompR*, and Δ *phoB* Δ *ompR*, almost disrupted the adaptive growth. We examined whether double RR gene knockout of other combinations. Cluster analysis with all RRs of *E. coli* K-12 showed that PhoP, PhoB, and OmpR were highly similar RRs (Fig. S2). Therefore, we knocked out a pair of similar genes and isolated four double RR knockout strains of Δ *phoP* Δ *kdpE*, Δ *phoB* Δ *creB*, Δ *ompR* Δ *cpxR*, and Δ *ompR* Δ *rstA* (see above) (Fig. S4). Most of the cells of the Δ *phoP* Δ *kdpE*, Δ *phoB* Δ *creB*, Δ *ompR* Δ *cpxR*, and Δ *ompR* Δ *rstA* strains grew significantly at the end of observation after 8 hours (Fig. S6). Three double RR gene knockout strains, Δ *phoP* Δ *kdpE*, Δ *phoB* Δ *creB*, and Δ *ompR* Δ *cpxR*, showed an elapsed time in the range of 2.5–4.0 hours, and the growth rate varied in the range of 0.75–1.5 divisions/hour (Figs. 3D, S6 and S7). On the other hand, Δ *ompR* Δ *rstA* strains showed an elapsed time in the range of 2.5–7.5 hours, and the growth rate varied in the range of 0.05–1.2 divisions/hour (Figs. 3D, S6 and S7). Furthermore, another triple RR gene knockout strain, Δ *rstA* Δ *cusR* Δ *hrpR*, also presented micro-colony formation in 92.6% of the population, starting cell division 1.5–4.5 hours after incubation (83.9%) and showing a growth rate of 0.6–2.0 divisions/hour (69.6%), as did the Δ *phoQ* Δ *phoR* Δ *envZ* strain (Figs. 3C, S6w and S7).

We next examined three cognate pair double gene knockout strains, Δ *phoP* Δ *phoQ*, Δ *phoB* Δ *phoR*, and Δ *ompR* Δ *envZ*, for adaptive growth. Most of the cells of the three cognate pair double gene knockout strains grew significantly at the end of observation after 8 hours (Fig. S6). These three strains showed an elapsed time in the range of 1.0–3.5 hours, but their growth rates varied (Figs. 3B and S6, S7). The Δ *phoP* Δ *phoQ* and Δ *ompR* Δ *envZ* strains showed growth rates in the range of 1.3–2.7 divisions/hour (70%) and 1.3–2.7 divisions/hour (70%), respectively, whereas 70% of cells of the Δ *phoB* Δ *phoR* strain showed growth rates of 0.4–1.4 divisions/hour, similar to the parent strain (Figs. 3B and S7). In agreement with observations of single knockout strains as shown above, these observations showed that the roles of PhoP, PhoB, and OmpR did not completely correspond with their cognate SKs, PhoQ, PhoR, and EnvZ, respectively, in adaptive growth.

To demonstrate the genetic involvement of TCSs for adaptive growth, cluster analysis was performed with the elapsed time and the growth rate on solid medium (Fig. S8). The dendrogram of the elapsed time showed two groups, each divided into two sub-groups (Fig. S8a). The parent strain was assigned to Group-Ib, which included not only all single RR gene knockout strains but also all double SK gene knockout strains and triple RR and SK gene knockout strains (Fig. S8a). Additionally, all cognate pair gene knockout strains were assigned to Group-Ia (Fig. S8a). All of the single SK gene knockout strains were assigned to Group II, in which all of the double RR gene knockout strains were part of Group IIb (Fig. S8a). The dendrogram of the growth rate also showed two groups (Fig. S8b). The parent strain was assigned to Group II, which included most of the knockout strains, whereas all of the double RR gene knockout strains were isolated in Group I (Fig. S8b).

Cluster analysis of the elapsed time also showed that all four of the double RR gene knockout strains studied were assigned to Group-Ia but not to Group-II (Fig. S8a). Additionally, clustering for the growth rate showed that all four of the double RR gene knockout strains were not assigned to Group I (Fig. S8b). Taken together, knockout of the double RR gene among *ompR*, *phoP*, and *phoB*, specifically caused a long delay in the start of growth and a lower growth rate (Fig. 3).

## Discussion

Bacteria are unicellular organisms. Individual cells are known to start growth under rich nutrient conditions by operating metabolic networks. On the other hand, bacterial cells that survive have a proper memory for the function of genetic stress response systems. Thus, multi-genetic factors function for bacterial growth. To analyse the function of several genes, we developed the CRISPR-Cas9-based HoSeI method to knock out genes without antibiotic resistance gene markers. The HoSeI method is a genetic marker-less genome editing approach and introduces base substitutions in the target sequence on the original genome by screening dead or alive cells. We repeated the HoSeI method in the* E. coli* K-12 genome and isolated multi-gene knockout strains. This should be a powerful method to edit genome sequences, e.g., knockout of multiple genes and artificial introduction of mutations, which are useful experimental demonstrations of bacterial genome-wide epistatic phenomena.

**Figure 4:**
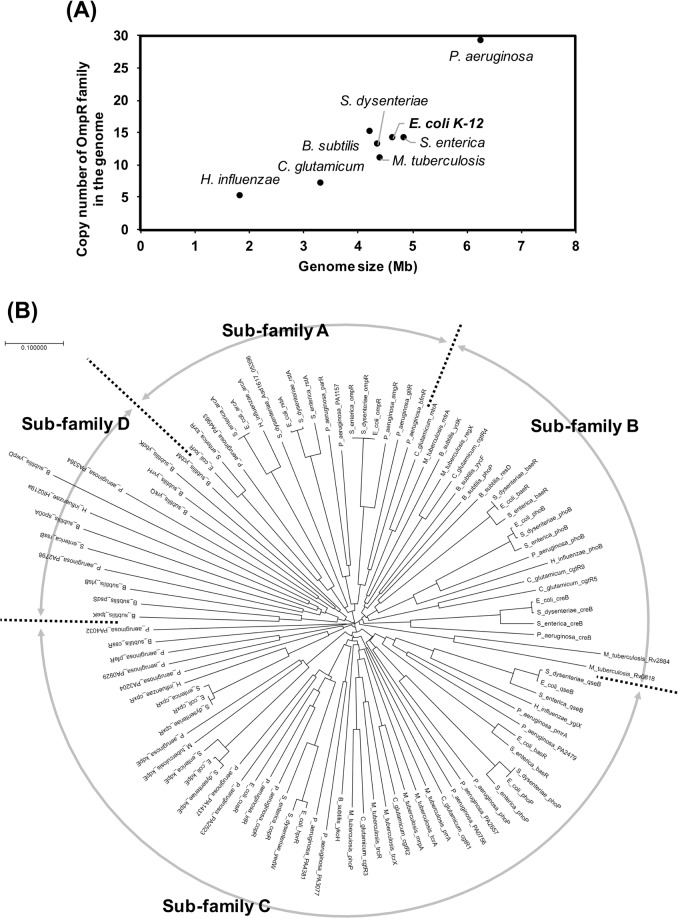
Comparison of OmpR family RRs among several bacteria. (**A**) OmpR family RR gene copy number of several bacteria. On the basis of the data shown in Fig. 1, the copy number of OmpR family RR genes (y-axis) in eight bacterial genomes was compared with the respective genome size (x-axis). The eight bacterial strains are *Haemophilus influenzae* Rd KW20, *Corynebacterium glutamicum* ATCC 13032, *Bacillus subtilis* subsp. subtilis str. 168, *Mycobacterium tuberculosis* H37Rv, *Shigella dysenteriae* 1617, *Escherichia coli* str. K-12 substr. MG1655, *Salmonella enterica* subsp. enterica serovar Typhimurium str. LT2, and *Pseudomonas aeruginosa* PAO1. (**B**) Phylogenetic analysis of the amino acid sequence of OmpR family RRs. All of the amino acid sequences of the OmpR family RRs in 8 bacteria shown in (**A**) were compared and analysed with ClustalW software. The result divided the RRs into four sub-families. The amino acid sequence of the used OmpR family RRs was compiled in Table S4.

An OmpR family comparison of *Haemophilus influenzae* Rd KW20, *Corynebacterium glutamicum* ATCC 13032, *Bacillus subtilis* subsp. subtilis str. 168, *Mycobacterium tuberculosis* H37Rv, *Shigella dysenteriae* 1617, *Escherichia coli* str. K-12 substr. MG1655, *Salmonella enterica* subsp. enterica serovar Typhimurium str. LT2, and *Pseudomonas aeruginosa* PAO1 reveals that the four sub-families actually divided PhoP, PhoB, and OmpR (Fig. 4B and Table S4). OmpR family proteins of *E. coli* were classified into 3 sub-families, except for sub-family D, and PhoP, PhoB, and OmpR were divided into sub-families C, B, and A, respectively. The result of the OmpR family comparison was mostly identical to the result of cluster analysis with all RRs of *E. coli* K-12 (Figs. 4B and S2A). Additionally, OmpR family RRs are conserved not only in closely related bacteria with genome sizes similar to that of *E. coli* but also in bacteria with smaller (*H. influenzae*) or larger (*P. aeruginosa*) genomes than that of *E. coli*. A genome comparison of *E. coli* strains reveals that the predicted pan-genome comprises 15,741 gene families and that the core genome comprises only 993 (6%) of the families, as represented in every genome^25^. OmpR family genes are included in the *E. coli* core genome and increase their number in the genome. We found that the OmpR family includes conserved TCS genes withing the bacterial genome. The number of the OmpR family gene is also correlated with the size of the genome (Fig. 4A). The *E. coli* K-12 genome has 14 genes of the OmpR family, occupying more than 40% of the total number of RR genes. The intracellular levels of 65 species of transcription factors with known function in *E. coli* K-12 W3110 type A at various phases of cell growth showed the following decreasing order for the intracellular response regulators: PhoP -> PhoB -> CitB -> OmpR -> NarL -> CpxR -> UhpA -> RstA -> EvgA -> KdpE -> TorR -> UvrY -> QseB -> ArcA^20^. Among these 14 more abundant species, 9 species of RRs belong to the OmpR family (PhoP, PhoB, OmpR, CpxR, RstA, KdpE, TorR, QseB, and ArcA). We isolated all of 14 OmpR family single RR gene knockout strains by HoSeI method, indicating that none of them was essential for growth under the used conditions in good agreement with previous works^26,27^. Time-lapse live cell imaging with microscope indicated that 14 single RR gene knockout strains were divided into three groups, the faster growth rate mutants, the normal growth rate mutants, and the slow growth rate mutant (Fig. 2B). The difference did not appear to correlate with the intracellular level or the number of regulated genes. Despite having different initial growth times, all single RR gene knockout strains showed similar growth curves in liquid M9 glucose and LB media, as did the parent strain (data not shown). These results suggest that each RR gene could cause fluctuations of the initial growth rate in the range of 0.5–2.0 divisions/hour among the bacterial populations. However, it is unclear how each RR gene changes the homogeneity of the initial growth rate in the bacterial population.

As unexpected, we found that knockout of the arbitrary pair of *phoP, phoB*, and *ompR* are shown as an epistatic inhibition for *E. coli* adaptive growth (Fig. 3). When the species of three genes knockout was introduced into single *phoP* knockout strain (Table S1), only double defect with *ompR* gene dramatically inhibited the adaptive growth of *E. coli* (Fig. 3). When the species of four genes knockout was introduced into single *phoB* knockout strain (Table S1), only double defect with either *ompR* or *phoP* gene dramatically inhibited the adaptive growth of *E. coli* (Fig. 3). Besides, the knockout for *citB* gene, one of NarL family RR gene of *E. coli*, was introduced into both single *phoB* and single *phoP* knockout strains, resulting that both double gene knockout strains, Δ *citB* Δ *phoB* and Δ *citB* Δ *phoP*, did not show significant inhibition of the adaptive growth of *E. coli* (data not shown). Despite different genetic backgrounds, the genetic defect of an arbitrary pair of *phoP, phoB*, and *ompR* largely delays initiation of growth and dramatically reduces the initial growth rate, the results of which were entirely different from not only *phoP, phoB*, and *ompR* RR single knockouts but also their triple knockouts (Figs. 3 and S8). In good agreement with the most abundant RRs, PhoP, PhoB, and OmpR^20^, a memory of expression of genes below cytotoxic levels exists for positively autoregulated systems of the genes *phoP, phoB*, and *ompR* in *E. coli*. Each OmpR family member, including *E. coli* PhoP, PhoB, and OmpR, contains an N-terminal receiver domain (RD) and a C-terminal DNA-binding domain (DBD), which are connected by a linker. To date, the molecular structure of the truncated RD and DBD and the full-length active and inactive form of OmpR family proteins have been solved by X-ray crystallography and NMR spectroscopy^28–36^. Among these proteins, dimerization or higher-order multimerization is commonly observed, and at least three surfaces have been observed for intermolecules. Two surfaces, the *α*1*α*5 and the *α*4*β*5*α*5 faces, conserve the RD and one surface the DBD. PhoP, PhoB, and OmpR are known to bind to at least 15 promoters *in vivo*, as denoted in RegulonDB (http://regulondb.ccg.unam.mx/index.jsp), indicating that the intracellular level of approximately 1,000 copies of PhoP, PhoB, and OmpR each per genome is clearly too high, and thus, these proteins could play a role except in the stress response. These are in good agreement with the observation of the adaptive growth of *E. coli* of a knockout of an arbitrary pair of *envZ, phoR*, and *phoQ* genes, the cognate SK genes for OmpR, PhoB, and PhoP (Figs. 3C, S6w and S7). PhoB and OmpR are also known to bind to more than 100 genomic sites *in vitro* denoted in TEC (https://shigen.nig.ac.jp/E.coli/tec/top/), suggesting that PhoP, PhoB, and OmpR each basically function as a DNA-binding protein. Genome-wide binding profiles have indicated the recognized DNA sequences TTTAnnnnTTTA as the PhoP-binding consensus^37^, TTTGTTACAT as the OmpR-binding consensus^38^, and TGTnAnAAAnnTGTnA as the PhoB-binding consensus^39^. The tandem triplet of A or T, found as a common sequence among the genes, could be weakly recognized by every one of PhoB, PhoP, and OmpR. Thus, *E. coli* cells could show adaptive growth when a total of 3,000 intracellular proteins of PhoP, PhoB, and OmpR have become associated with the genome. *E. coli* cells, however, could not properly grow, if insignificant occupation of the genome by 1,000 intracellular proteins of either PhoP, PhoB, or OmpR.

The triple RR gene knockout strain, however, recovered the elapsed time and the growth rate to values similar to those for the parent strain (Fig. 2). This observation showed that a total of 3,000 intracellular proteins of PhoP, PhoB, and OmpR was not essential for adaptive growth of *E. coli*. One possible reason that normal growth of the triple RR gene knockout strain is the existence of excess intracellular RRs in place of PhoP, PhoB, and OmpR. We did not, however, find a significant difference in the profile of cellular proteins, with more than 200 proteins detected with LC-MS, between the parent strain and the triple RR gene knockout strain (data not shown). Additionally, the promoter activities of all TCS genes in the *E. coli* genome were measured using the luciferase reporter system, indicating that three double RR gene knockout strains and a triple RR gene knockout strain were mostly similar to the parent strain with respect to these promoter activities (data not shown).

These findings suggest that the mode of action for inhibition of adaptive growth by knockout of the arbitrary pair of *phoP*, *phoB*, and *ompR* is important for the bacterial cell cycle, genome replication and/or cell division but not for general metabolism and stress responses. The epistatic inhibition for *E. coli* adaptive growth by an arbitrary pair of *phoP, phoB*, and *ompR* was obscure on LB agar (data not shown). All of the isolated knockout strains also showed a similar adaptive growth to the parent strain on LB agar (data not shown). One possible is that only either PhoP, PhoB, or OmpR at a high intracellular level bias genome expression profile and inhibit the optimum adaptive growth in *E. coli* grown in M9 glucose that contains less amino acids than LB broth. Another is that a high concentration of only one species of OmpR family severely affect bacterial growth by an unknown molecular mechanism, which could be suppressed by a high concentration of another species of OmpR family. These findings suggest that RRs contribute to the optical high level of the intracellular RRs, resulting in a fitness advantage for growth. The results are in good agreement with the idea of fitness advantages being conferred for both positive and negative feedback in both dynamic and stable environments. Taken together, in *E. coli*, PhoP, PhoB, and OmpR are autoregulated at the optimal intracellular level and balance of their intracellular level play an important role in survival for repeatable growth fitness under the evaluated experimental conditions.

## Methods

### *E. coli* strains, plasmids, and oligonucleotides

The used *E. coli* strains, plasmids, and oligonucleotides are listed in Tables S1, S2 and S3, respectively. *E. coli* K-12 W3110 typeA strain was used as a parent type^40^.

### Growth condition of *E. coli*

*E. coli* cells were grown at 30 °C in M9-glucose medium.

### Construction of sgRNA expression plasmid

To construct the sgRNA expression vector plasmid, 227 bp of the DNA sequence of the promoter and sgRNA was referred to as the pTarget series harbouring sgRNAs constructed by previous work^21^. The designed 227 bp DNA sequence introducing the *Not* I recognition site immediately upstream of tracrRNA (trans-activating CRISPR RNA) was synthesized and cloned into the pEX-A2 vector by Eurofin genomics (Tokyo, Japan), resulting in the construction of psgRNA (Table S2). Insertion DNA for the target sequence was prepared by hybridization of a pair of complementary synthetic oligonucleotides in length of 50-nt each: ompR_sgRNA_N20 and ompR_sgRNA_com for the *ompR* gene; phoP_sgRNA_N20 and phoP_sgRNA_com for the *phoP* gene; phoB_sgRNA_N20 and phoB_sgRNA_com for the *phoB* gene; envZ_sgRNA_N20 and envZ_sgRNA_com for the *envZ* gene; phoR_sgRNA_N20 and phoR_sgRNA_com for the *phoR* gene; phoQ_sgRNA_N20 and phoQ_sgRNA_com for the *phoQ* gene; kdpE_sgRNA_N20 and kdpE_sgRNA_com for the *kdpE* gene; creB_sgRNA_N20 and creB_sgRNA_com for the *creB* gene; cpxR_sgRNA_N20 and cpxR_sgRNA_com for the *cpxR* gene; rstA_sgRNA_N20 and rstA_sgRNA_com for the *rstA* gene; cusR_sgRNA_N20 and cusR_sgRNA_com for the *cusR* gene; hprR_sgRNA_N20 and hprR_sgRNA_com for the *hprR* gene; arcA_sgRNA_N20 and arcA_sgRNA_com for the *arcA* gene; basR_sgRNA_N20 and basR_sgRNA_com for the *basR* gene; baeR_sgRNA_N20 and baeR_sgRNA_com for the *baeR* gene; qseB_sgRNA_N20 and qseB_sgRNA_com for the *qseB* gene; and torR_sgRNA_N20 and torR_sgRNA_com for the *torR* gene (see DNA sequence shown in Table S3). The DNA was inserted into the *Not* I site of psgRNA vector by the In-Fusion system (Takara Bio, Japan). The inserted DNA sequence on the resulting plasmids was confirmed by DNA sequencing (Table S3).

### Multi-gene knockout in the *E. coli* genome by the HoSeI method based on CRISPR-Cas9

The W3110 typeA strain harbouring pCas was grown in LB medium containing 1% arabinose and 50 *μ*g/mL kanamycin to logarithmic phase and then was collected and suspended in a solution of 0.1 M CaCl_2_. This suspension of *E. coli* was subjected to transformation by the psgRNA-target and DNA fragment to recover the digested site by CRISPR-Cas9. psgRNA-ompR, psgRNA-phoB, psgRNA-phoP, psgRNA-envZ, psgRNA-phoR, psgRNA-phoQ, psgRNA-kdpE, psgRNA-creB, psgRNA-cpxR, psgRNA-rstA, psgRNA-cusR, psgRNA-hprR, psgRNA-arcA, psgRNA-basR, psgRNA-baeR, psgRNA-qseB, and psgRNA-torR were used as psgRNA-target plasmids (Table S2). The 83-bp DNA fragments were prepared by hybridization of a pair of complementary synthetic oligonucleotides: ompR_PAMstop and ompR_PAMstop_com for the *ompR* gene; phoP_PAMstop and phoP_PAMstop_com for the *phoP* gene; phoB_PAMstop and phoB_PAMstop_com for the *phoB* gene; envZ_PAMstop and envZ_PAMstop_com for the *envZ* gene; phoR_PAMstop and phoR_PAMstop_com for the *phoR* gene; phoQ_PAMstop and phoQ_PAMstop_com for the *phoQ* gene; kdpE_PAMstop and kdpE_PAMstop_com for the *kdpE* gene; creB_PAMstop and creB_PAMstop_com for the *creB* gene; cpxR_PAMstop and cpxR_PAMstop_com for the *cpxR* gene; rstA_PAMstop and rstA_PAMstop_com for the *rstA* gene; cusR_PAMstop and cusR_PAMstop_com for the *cusR* gene; hprR_PAMstop and hprR_PAMstop_com for the *hprR* gene; arcA_PAMstop and arcA_PAMstop_com for the *arcA* gene; basR_PAMstop and basR_PAMstop_com for the *basR* gene; baeR_PAMstop and baeR_PAMstop_com for the *baeR* gene; qseB_PAMstop and qseB_PAMstop_com for the *qseB* gene; and torR_PAMstop and torR_PAMstop_com for the *torR* gene (see the DNA sequences shown in Table S3). In comparison with no colonies observed on LB agar containing 100 *μ*g/mL ampicillin by transformation with only psgRNA-target, the addition of the DNA fragment produced transformants that grew on LB agar containing ampicillin. To verify the introduction of a stop codon on the target gene of the genome, genomic DNA was prepared from the transformant and used as a template for amplification of the target sequence by PCR using a pair of oligonucleotides, as shown in Table S3. The introduction of a stop codon on the target gene was confirmed by DNA sequencing of the amplified DNA.

### Time-lapse observation on microscope

Strains were inoculated in M9 glucose medium and shaken overnight at 37 °C. The cultures were washed with M9 medium three times and diluted 10-fold into the medium. The diluted cultures were spread on an M9 glucose plate. Next, the plates were cut and placed on a slide glass. The preparation was sealed on a glass coverslip with nail polish. The cells were imaged with a microscope (IX81, Olympus) using a 100x/NA 1.4 objective lens (M Plan Apochromat MPLAPON-Oil, Olympus) and a Retiga EXi Fast1394 CCD camera (Q Imaging). The temperature was maintained at 37 °C using a closed circulation system (EYELA). Image acquisition and microscope control were performed with Image Pro Plus (Nippon roper). The elapsed time until cell division and growth rate of each *E. coli* strain were measured by ImageJ.

### Construction of RR protein expression plasmid

We constructed the OmpR, PhoB, and PhoP expression plasmids as well as arabinose-inducible expression system of NarL family RR as previously described^41^. In brief, the protein-coding regions of *phoP*, *phoB*, and *ompR* gene with an artificial SD sequence were amplified by PCR using specific primers (Table S3). Each amplified DNA fragment was inserted into the linear pBAD33 vector by In Fusion system (Takara Bio, Japan), resulting in construction of the FLAG-tagged RR protein expression plasmids, pBADPhoP-FLAG, pBADPhoB-FLAG, and pBADOmpR-FLAG (Table S2).

### Luciferase reporter assay in *E. coli*

We performed luciferase reporter assay in *E. coli* to evaluate activity of TCS-target promoters *in vivo* as previously described^42,43^. In brief, the *ompC, mgtA, pstS* promoters were amplified by PCR using W3110 type A genome as a template, a pair of primers (see DNA sequence shown in Table S3), and Ex Taq DNA polymerase (Takara Bio, Japan). The promoter DNA was inserted into the *Bam* HI and *Xho* I sites of pLUX vector^44^ by the In-Fusion system (Takara Bio, Japan). The inserted DNA sequence on the resulting plasmid was confirmed by DNA sequencing (Table S2). The constructed pLux-mgtA, pLux-pstS, and pLux-ompC were transformed into each strain. Transformants were grown in the M9-glucose medium at 37 °C with shaking for overnight. Then, overnight culture was transferred into fresh M9-glucose medium and OD_600_ and a total intensity of luminescence of culture were measured with a plate reader (Corona). The ratio of luminescence to OD_600_ (LUX/OD) was evaluated as a specific activity of the promoter.

### Cluster analysis

Cluster analysis was performed with R software. The obtained data of the elapsed time and the growth rate were prepared for Ward’s method. A hierarchical clustering dendrogram was calculated using Euclidean distances.

### Correlation of the number of COGs and the genome size with the Gompertz function

We used all of the genome sequences of 628 species of bacteria registered in the COG databank. TCS genes and RNA polymerase genes were isolated, and the number of these genes was counted for each bacterial genome. The number of COGs involved in TCSs and the RNA polymerase subunit was analysed in comparison with the genome size, and the average number of COGs was calculated for each genome size of 0.5, 1, 2, 3, 4, 5, 6, 7, 8, 9, 10, 12, and 15 Mb. The calculated data were fitted by the Gompertz curve $y=a{b}^{{e}^{-cx}}$, where y is the number of COGs and x is the genome size.

## Supplementary information

Supplementary Information.
