# Supplementary material for: Epistatic Effect of Regulators to the Adaptive Growth of Escherichia coli
Source: Sci Rep. 2020 Feb 27;10:3661. doi: 10.1038/s41598-020-60353-3 (PMC7046781; doi:10.1038/s41598-020-60353-3)
Supplement: Supplementary file 1 — Supplementary Information. [file 41598_2020_60353_MOESM1_ESM.pdf]

**Supplemental Information**

# Epistatic effect of Regulators to the Adaptive Growth of *Escherichia coli*

Yukari Miyake and Kaneyoshi Yamamoto
Hosei Univeristy, Department of Frontier Bioscience and Research Institute of Micro-Nano Technology, Koganei, Tokyo 184-8584, Japan

**Contents:**

**Table S1. Bacterial strains used in this study.**
**Table S2. Plasmids used in this study.**
**Table S3. Oligonucleotides used in this study.**
**Table S4. The amino acid sequence of OmpR family RRs.**
**Figure S1. The correlation between genome size and the number of RNA polymerase subunit on bacterial genome.**
**Figure S2. Classification of two-component system RRs in *E. coli.***
**Figure S3. Homologous sequence integration (HoSeI) method for multi-gene knockout on *E. coli* genome.**
**Figure S4. Construction of RR or SK-coding gene knockout on *E. coli* genome.**
**Figure S5. Single cell analysis of adaptive growth of single RR gene knockout *E. coli* strain.**
**Figure S6. Single cell analysis of adaptive growth of multi-gene knockout *E. coli* strain.**
**Figure S7. The distribution of cell growth values of *E. coli* strains.**
**Figure S8. Cluster analysis using values for *E. coli* cell growth of strains.**
**References**

Table S1. Bacterial strains used in this study.

| Name | Characterization | Reference |
|---|---|---|
| W3110 typeA | Wild type, complete σ set | Jishage, et al., 1997 |
| W3110ΔompR | W3110, Δ*ompR* (W3110 typeA←Δ*ompR*) | This study |
| W3110ΔphoB | W3110, Δ*phoB* (W3110 typeA←Δ*phoB*) | This study |
| W3110ΔphoP | W3110, Δ*phoP* (W3110 typeA←Δ*phoP*) | This study |
| W3110ΔkdpE | W3110, Δ*kdpE* (W3110 typeA←Δ*kdpE*) | This study |
| W3110ΔcusR | W3110, Δ*cusR* (W3110 typeA←Δ*cusR*) | This study |
| W3110ΔrstA | W3110, Δ*rstA* (W3110 typeA←Δ*rstA*) | This study |
| W3110ΔhprR | W3110, Δ*hprR* (W3110 typeA←Δ*hprR*) | This study |
| W3110ΔcreB | W3110, Δ*creB* (W3110 typeA←Δ*creB*) | This study |
| W3110ΔarcA | W3110, Δ*arcA* (W3110 typeA←Δ*arcA*) | This study |
| W3110ΔbasR | W3110, Δ*basR* (W3110 typeA←Δ*basR*) | This study |
| W3110ΔbaeR | W3110, Δ*baeR* (W3110 typeA←Δ*baeR*) | This study |
| W3110ΔqseB | W3110, Δ*qseB* (W3110 typeA←Δ*qseB*) | This study |
| W3110ΔtorR | W3110, Δ*torR* (W3110 typeA←Δ*torR*) | This study |
| W3110ΔcpxR | W3110, Δ*cpxR* (W3110 typeA←Δ*cpxR*) | This study |
| W3110ΔompRΔphoB | W3110, Δ*ompR,* Δ*phoB* (W3110ΔphoB←Δ*ompR*) | This study |
| W3110ΔompRΔphoP | W3110, Δ*ompR,* Δ*phoP* (W3110ΔphoP←Δ*ompR*) | This study |
| W3110ΔphoBΔphoP | W3110, Δ*phoB,* Δ*phoP* (W3110ΔphoB←Δ*phoP*) | This study |
| W3110ΔompRΔphoBΔphoP | W3110, Δ*ompR*, Δ*phoB,* Δ*phoP* (W3110ΔphoBΔphoP←Δ*ompR*) | This study |
| W3110ΔompRΔenvZ | W3110, Δ*ompR,* Δ*envZ* (W3110ΔompR←Δ*envZ*) | This study |
| W3110ΔphoBΔphoR | W3110, Δ*phoB,* Δ*phoR* (W3110ΔphoB←Δ*phoR*) | This study |
| W3110ΔphoPΔphoQ | W3110, Δ*phoP,* Δ*phoQ* (W3110ΔphoP←Δ*phoQ*) | This study |
| W3110ΔphoPΔkdpE | W3110, Δ*phoP,* Δ*kdpE* (W3110ΔphoP←Δ*kdpE*) | This study |
| W3110ΔphoBΔcreB | W3110, Δ*phoB,* Δ*creB* (W3110ΔphoB←Δ*creB*) | This study |
| W3110ΔompRΔcpxR | W3110, Δ*ompR,* Δ*cpxR* (W3110ΔompR←Δ*cpxR*) | This study |
| W3110ΔompRΔrstA | W3110, Δ*ompR,* Δ*rstA* (W3110ΔompR←Δ*rstA*) | This study |
| W3110ΔrstAΔcusR | W3110, Δ*rstA,* Δ*cusR* (W3110ΔrstA←Δ*cusR*) | This study |
| W3110ΔrstAΔcusRΔhprR | W3110, Δ*rstA*, Δ*cusR*, Δ*hprR* (W3110ΔrstAΔcusR←Δ*hprR*) | This study |
| W3110ΔenvZ | W3110, Δ*envZ* (W3110 typeA←Δ*envZ*) | This study |
| W3110ΔphoR | W3110, Δ*phoR* (W3110 typeA←Δ*phoR*) | This study |
| W3110ΔphoQ | W3110, Δ*phoQ* (W3110 typeA←Δ*phoQ*) | This study |
| W3110ΔenvZΔphoR | W3110, Δ*envZ,* Δ*phoR* (W3110ΔphoR←Δ*envZ*) | This study |

| | | |
|---|---|---|
| W3110ΔenvZΔphoQ | W3110, Δ*envZ, ΔphoQ* (W3110ΔphoQ←Δ*envZ*) | This study |
| W3110ΔphoRΔphoQ | W3110, Δ*phoR, ΔphoQ* (W3110ΔphoQ←Δ*phoR*) | This study |
| W3110ΔenvZΔphoRΔphoQ | W3110, Δ*envZ*, Δ*phoR, ΔphoQ* (W3110ΔphoQΔphoR←Δ*envZ*) | This study |

The *E. coli* strains constructed in this study would be provided from National BioResource Project (NBRP) *E. coli* of Japan.

Table S2. Plasmids used in this study.

| Name | Characterization | Reference |
|---|---|---|
| pCas | *repA*$^{ts}$ *kan* $P_{cas}$-*cas9* $P_{araB}$-*red lacI*$^{q}$ $P_{trc}$-sgRNA-pMB1 | Jiang et al., 2015 |
| psgRNA | pMB1 *bla* P-sgRNA (sgRNA cloning vector) | This study |
| psgRNA-ompR | pMB1 *bla* P-sgRNA-*ompR* | This study |
| psgRNA-phoB | pMB1 *bla* P-sgRNA-*phoB* | This study |
| psgRNA-phoP | pMB1 *bla* P-sgRNA-*phoP* | This study |
| psgRNA-kdpE | pMB1 *bla* P-sgRNA-*kdpE* | This study |
| psgRNA-creB | pMB1 *bla* P-sgRNA-*creB* | This study |
| psgRNA-cpxR | pMB1 *bla* P-sgRNA-*cpxR* | This study |
| psgRNA-rstA | pMB1 *bla* P-sgRNA-*rstA* | This study |
| psgRNA-hprR | pMB1 *bla* P-sgRNA-*hprR* | This study |
| psgRNA-arcA | pMB1 *bla* P-sgRNA-*arcA* | This study |
| psgRNA-basR | pMB1 *bla* P-sgRNA-*basR* | This study |
| psgRNA-baeR | pMB1 *bla* P-sgRNA-*baeR* | This study |
| psgRNA-qseB | pMB1 *bla* P-sgRNA-*qseB* | This study |
| psgRNA-torR | pMB1 *bla* P-sgRNA-*torR* | This study |
| psgRNA-cusR | pMB1 *bla* P-sgRNA-*cusR* | This study |
| psgRNA-envZ | pMB1 *bla* P-sgRNA-*envZ* | This study |
| psgRNA-phoR | pMB1 *bla* P-sgRNA-*phoR* | This study |
| psgRNA-phoQ | pMB1 *bla* P-sgRNA-*phoQ* | This study |
| pLUX | *kan*, *luxCDABE*, STOP codons, ribosome binding site | Burton NA. et al., 2010 |
| pLux-mgtA | pLUX, *mgtA* promoter-*luxCDABE* | This study |
| pLux-pstS | pLUX, *pstS* promoter-*luxCDABE* | This study |
| pLux-ompC | pLUX, *ompC* promoter-*luxCDABE* | This study |
| pBAD33 | pACYC184 derived, $P_{BAD}$ $Cm^{r}$ | Guzman et al. 1995 |
| pBADPhoP-FLAG | pBAD33, FLAG-tagged PhoP at C-terminus | This study |
| pBADPhoB-FLAG | pBAD33, FLAG-tagged PhoB at C-terminus | This study |
| pBADOmpR-FLAG | pBAD33, FLAG-tagged OmpR at C-terminus | This study |

The plasmids constructed in this study would be provided from National BioResource Project (NBRP) *E. coli* of Japan.

Table S3. Oligonucleotides used in this study.

| Name | Sequence (5' to 3') |
|---|---|
| For psgRNA cloning in HoSeI system | |
| ompR_sgRNA_N20 | CCGCAGCGGTTTCAGAACGTTCCAGCAGCGCACGCGTTTTAGAGCTAGAA |
| ompR_sgRNA_com | TTCTAGCTCTAAAACGCGTGCGCTGCTGGAACGTTCTGAAACCGCTGCGG |
| phoP_sgRNA_N20 | CCGCAGCGGTTTCAGGATATTGCGATTGTCGATCTGTTTTAGAGCTAGAA |
| phoP_sgRNA_com | TTCTAGCTCTAAAACAGATCGACAATCGCAATATCCTGAAACCGCTGCGG |
| phoB_sgRNA_N20 | CCGCAGCGGTTTCAGCGAACAAAATGGCTTTCAGCGTTTTAGAGCTAGAA |
| phoB_sgRNA_com | TTCTAGCTCTAAAACGCTGAAAGCCATTTTGTTCGCTGAAACCGCTGCGG |
| rstA_sgRNA_N20 | CCGCAGCGGTTTCAGTTCACTGATTGCCGCGTACCGTTTTAGAGCTAGAA |
| rstA_sgRNA_com | TTCTAGCTCTAAAACGGTACGCGGCAATCAGTGAACTGAAACCGCTGCGG |
| cpxR_sgRNA_N20 | CCGCAGCGGTTTCAGTTAATAGGGAAGTCAGCTCTGTTTTAGAGCTAGAA |
| cpxR_sgRNA_com | TTCTAGCTCTAAAACAGAGCTGACTTCCCTATTAACTGAAACCGCTGCGG |
| creB_sgRNA_N20 | CCGCAGCGGTTTCAGGCAAGGGATAGCCGACACGCGTTTTAGAGCTAGAA |
| creB_sgRNA_com | TTCTAGCTCTAAAACGCGTGTCGGCTATCCCTTGCCTGAAACCGCTGCGG |
| kdpE_sgRNA_N20 | CCGCAGCGGTTTCAGTATTCGTCGCTTTCTGCGCAGTTTTAGAGCTAGAA |
| kdpE_sgRNA_com | TTCTAGCTCTAAAACTGCGCAGAAAGCGACGAATACTGAAACCGCTGCGG |
| cusR_sgRNA_N20 | CCGCAGCGGTTTCAGACCGGAGAATACTTGACCAAGTTTTAGAGCTAGAA |
| cusR_sgRNA_com | TTCTAGCTCTAAAACTTGGTCAAGTATTCTCCGGTCTGAAACCGCTGCGG |
| hprR_sgRNA_N20 | CCGCAGCGGTTTCAGTGAAGATAATCAAAGGACCCGTTTTAGAGCTAGAA |
| hprR_sgRNA_com | TTCTAGCTCTAAAACGGGTCCTTTGATTATCTTCACTGAAACCGCTGCGG |
| arcA_sgRNA_N20 | CCGCAGCGGTTTCAGGTTGAAAAGTATTTTCGAAGGTTTTAGAGCTAGAA |
| arcA_sgRNA_com | TTCTAGCTCTAAAACCTTCGAAAATACTTTTCAACCTGAAACCGCTGCGG |
| basR_sgRNA_N20 | CCGCAGCGGTTTCAGATTCTGGCGGCGCAAACCGAGTTTTAGAGCTAGAA |
| basR_sgRNA_com | TTCTAGCTCTAAAACTCGGTTTGCGCCGCCAGAATCTGAAACCGCTGCGG |
| baeR_sgRNA_N20 | CCGCAGCGGTTTCAGTCTTCCACGATCAAAATACGGTTTTAGAGCTAGAA |

| | |
|---|---|
| baeR_sgRNA_com | TTCTAGCTCTAAAACCGTATTTTGATCGTGGAAGACTGAAACCGCTGCGG |
| qseB_sgRNA_N20 | CCGCAGCGGTTTCAGAGCGTCGACTGGTTTACACAGTTTTAGAGCTAGAA |
| qseB_sgRNA_com | TTCTAGCTCTAAAACTGTGTAAACCAGTCGACGCTCTGAAACCGCTGCGG |
| torR_sgRNA_N20 | CCGCAGCGGTTTCAGTGTTATTGTTGAAGATGAGCGTTTTAGAGCTAGAA |
| torR_sgRNA_com | TTCTAGCTCTAAAACGCTCATCTTCAACAATAACACTGAAACCGCTGCGG |
| envZ_sgRNA_N20 | CCGCAGCGGTTTCAGGACGATGAGCAATAACGTACGTTTTAGAGCTAGAA |
| envZ_sgRNA_com | TTCTAGCTCTAAAACGTACGTTATTGCTCATCGTCCTGAAACCGCTGCGG |
| phoR_sgRNA_N20 | CCGCAGCGGTTTCAGCACCCAGGATGAAAGCCGGGGTTTTAGAGCTAGAA |
| phoR_sgRNA_com | TTCTAGCTCTAAAACCCCGGCTTTCATCCTGGGTGCTGAAACCGCTGCGG |
| phoQ_sgRNA_N20 | CCGCAGCGGTTTCAGGTTTCGATAAAACTACGTTTGTTTTAGAGCTAGAA |
| phoQ_sgRNA_com | TTCTAGCTCTAAAACAAACGTAGTTTTATCGAAACCTGAAACCGCTGCGG |
| pEX_For | GGAGCAGACAAGCCCGTCAGG |
| pEX_Rev | CAGGCTTTACACTTTATGCTTCCGGC |

For DNA fragment preparation in HoSeI system

| | |
|---|---|
| ompR_PAMstop | AGAGAACTACAAGATTCTGGTGGTCGATGACGACATGCGCTAACGTGCGCTGCTGGAACGTTATCTCACCGAACAAGGCTTCC |
| ompR_PAMstop_com | GGAAGCCTTGTTCGGTGAGATAACGTTCCAGCAGCGCACGTTAGCGCATGTCGTCATCGACCACCAGAATCTTGTAGTTCTCT |
| phoP_PAMstop | TCTCAATGAACATATACCGGATATTGCGATTGTCGATCTCTAATTGCCAGACGAGGACGGTCTGTCACTGATTCGCCGCTGGC |
| phoP_PAMstop_com | GCCAGCGGCGAATCAGTGACAGACCGTCCTCGTCTGGCAATTAGAGATCGACAATCGCAATATCCGGTATATGTTCATTGAGA |
| phoB_PAMstop | CGAAATGGTCTGCTTCGTGCTCGAACAAAATGGCTTTCAGTAAGTCGAAGCGGAAGATTATGACAGTGCTGTGAATCAACTGA |
| phoB_PAMstop_com | TCAGTTGATTCACAGCACTGTCATAATCTTCCGCTTCGACTTACTGAAAGCCATTTTGTTCGAGCACGAAGCAGACCATTTCG |
| rstA_PAMstop | GGAAGATGATGCGGAAGTCGGTTCACTGATTGCCGCGTACTAAGCAAAACATGATATGCAGGTTACCGTAGAGCCGCGCGGCG |
| rstA_PAMstop_com | CGCCGCGCGGCTCTACGGTAACCTGCATATCATGTTTTGCTTAGTACGCGGCAATCAGTGAACCGACTTCCGCATCATCTTCC |
| cpxR_PAMstop | TATTTAAACAATGAATAAAATCCTGTTAGTTGATGATGACTAAGAGCTGACTTCCCTATTAAAGGAGCTGCTCGAGATGGAAG |
| cpxR_PAMstop_com | CTTCCATCTCGAGCAGCTCCTTTAATAGGGAAGTCAGCTCTTAGTCATCATCAACTAACAGGATTTTATTCATTGTTTAAATA |
| creB_PAMstop | GGTCTGGTTAGTGGAAGATGAGCAAGGGATAGCCGACACGTAAGTCTACATGTTGCAGCAGGAAGGTTTTGCCGTCGAGGTCT |

| | |
|---|---|
| creB_PAMstop_com | AGACCTCGACGGCAAAACCTTCCTGCTGCAACATGTAGACTTACGTGTCGGCTATCCCTTGCTCATCTTCCACTAACCAGACC |
| kdpE_PAMstop | GATTGTTGAAGATGAACAGGCTATTCGTCGCTTTCTGCGCTAAGCGCTGGAGGGCGACGGGATGCGCGTCTTTGAGGCCGAAA |
| kdpE_PAMstop_com | TTTCGGCCTCAAAGACGCGCATCCCGTCGCCCTCCAGCGCTTAGCGCAGAAAGCGACGAATAGCCTGTTCATCTTCAACAATC |
| cusR_PAMstop | TGTCGAAGATGAAAAGAAAACCGGAGAATACTTGACCAAATAATTAACCGAAGCCGGTTTTGTGGTCGATTTGGCCGACAACG |
| cusR_PAMstop_com | CGTTGTCGGCCAAATCGACCACAAAACCGGCTTCGGTTAATTATTTGGTCAAGTATTCTCCGGTTTTCTTTTCATCTTCGACA |
| hprR_PAMstop | TTTCATGAAGATTCTACTTATTGAAGATAATCAAAGGACCTAAGAATGGGTAACGCAGGGGCTTTCCGAAGCGGGTTATGTCA |
| hprR_PAMstop_com | TGACATAACCCGCTTCGGAAAGCCCCTGCGTTACCCATTCTTAGGTCCTTTGATTATCTTCAATAAGTAGAATCTTCATGAAA |
| arcA_PAMstop | CGAGTTGGTAACACGCAACACGTTGAAAAGTATTTTCGAATAAGAAGGCTATGATGTTTTCGAAGCGACAGATGGCGCGGAAA |
| arcA_PAMstop_com | TTTCCGCGCCATCTGTCGCTTCGAAAACATCATAGCCTTCTTATTCGAAAATACTTTTCAACGTGTTGCGTGTTACCAACTCG |
| basR_PAMstop | CGATACGCTGTTATTGCAGGGACTGATTCTGGCGGCGCAATAAGAAGGCTACGCGTGCGATAGCGTGACAACCGCGCGGATGG |
| basR_PAMstop_com | CCATCCGCGCGGTTGTCACGCTATCGCACGCGTAGCCTTCTTATTGCGCCGCCAGAATCAGTCCCTGCAATAACAGCGTATCG |
| baeR_PAMstop | AGAGAGAAGTATGACCGAGTTACCAATCGACGAAAACACATAACGTATTTTGATCGTGGAAGATGAACCGAAGCTGGGGCAGT |
| baeR_PAMstop_com | ACTGCCCCAGCTTCGGTTCATCTTCCACGATCAAAATACGTTATGTGTTTTCGTCGATTGGTAACTCGGTCATACTTCTCTCT |
| qseB_PAMstop | CCTTAGTAAAATGGGTTTTAGCGTCGACTGGTTTACACAATAACGTCAGGGAAAAGAGGCGCTTTATAGCGCACCTTATGATG |
| qseB_PAMstop_com | CATCATAAGGTGCGCTATAAAGCGCCTCTTTTCCCTGACGTTATTGTGTAAACCAGTCGACGCTAAAACCCATTTTACTAAGG |
| torR_PAMstop | CCCTCTGATGCCACATCACATTGTTATTGTTGAAGATGAGTAAGTTACCCAGGCGCGATTACAATCCTACTTCACTCAGGAGG |
| torR_PAMstop_com | CCTCCTGAGTGAAGTAGGATTGTAATCGCGCCTGGGTAACTTACTCATCTTCAACAATAACAATGTGATGTGGCATCAGAGGG |
| envZ_PAMstop | AAGCATGAGGCGATTGCGCTTCTCGCCACGAAGTTCATTTTAACGTACGTTATTGCTCATCGTCACCTTGCTGTTCGCCAGCC |
| envZ_PAMstop_com | GGCTGGCGAACAGCAAGGTGACGATGAGCAATAACGTACGTTAAAATGAACTTCGTGGCGAGAAGCGCAATCGCCTCATGCTT |
| phoR_PAMstop | GTCGTGGAAAAGGCTGGTGCTGGAGCTGCTACTTTGCTGCTAACCGGCTTTCATCCTGGGTGCATTTTTTGGTTACCTGCCCT |
| phoR_PAMstop_com | AGGGCAGGTAACCAAAAAATGCACCCAGGATGAAAGCCGGTTAGCAGCAAAGTAGCAGCTCCAGCACCAGCCTTTTCCACGAC |
| phoQ_PAMstop | GCTGATCGGTTATAGCGTCAGTTTCGATAAAACTACGTTTTAACTGTTACGTGGCGAGAGCAATCTGTTCTATACCCTTGCGA |
| phoQ_PAMstop_com | TCGCAAGGGTATAGAACAGATTGCTCTCGCCACGTAACAGTTAAAACGTAGTTTTATCGAAACTGACGCTATAACCGATCAGC |

For DNA amplification on *E. coli* genome in HoSeI system

| | |
|---|---|
| ompR_K306T | CAGCCAGATCTAGGAGGTTAAGACTCTTCC |

| | |
|---|---|
| ompR_check_R | GTCACCATAATGATCGGCATCGGGT |
| phoP_LL_F | TCGTCTTCACCTCGAAAGCAGTTTATCGATGGTCT |
| phoP_check_R | CTATTACGCCGCATTAATGCCTGCA |
| phoB_K030S | GAGGCAAGATCTGCGGCGCATGTCCGGCGC |
| phoB_check_R | TTTCAAGGCCGCGCACGCGATCTTC |
| rstA_LL_F | TCGTCTTCACCTCGAGCAAAGGTCGGGAAAAGTGG |
| rstA_check_R | GCTTTGTAGGGAGTCAGAGACGTTT |
| cpxR_K365T | CCCACAGATCTTGGGGGAAGACAGGGATGG |
| cpxR_check_R | GCATCAACTTCCAGTGTCGGTGAAC |
| creB_K420S | GCACCAGATCTCAGGTAATTGGATAATAGC |
| creB_check_R | TCACTTCGGGCCGTCAGGAACAGTA |
| kdpE_K425S | TCAGGCAGAGATCTTAACGCTCGATCTGGC |
| kdpE_check_R | TCGCTCTCTTCGCTGCGTGCGGAAA |
| cusR_K052T | GCGCATAGATCTGTTGCTGATTGAAATAGC |
| cusR_check_R | GCGGTAAGCAACAGAATCGGCATCC |
| hprR_K182T | TACATAGATCTGCATTAACTGCCACGATAG |
| hprR_check_R | CTTGCAGTAAGGCAAATAACAGGGG |
| arcA_K421T | CCATCGAGATCTTTGCCCGCGCCGCTGCCC |
| arcA_check_R | AGGAACATCAACGCAACATTCGCCT |
| basR_K389T | AGGCGAGATCTATATCATTCAGCGAGCGGG |
| basR_check_R | GCGATTTTGTCGGTCAGCGTATCGC |
| baeR_K190S | TGGGCAGATCTGCGCAAGACTTCAACCAGC |
| baeR_check_R | CGACGAATTTCCCGGCACAGCGTCA |
| qseB_K283S | TTCGCAGATCTGTAGTTGAGCAATTCAGGG |
| qseB_check_R | CACAGATAATCGTCAGCTCCCAGAC |
| torR_K097T | ACTGCAGATCTAAGCCGCTACTCATATCCG |

| | |
|---|---|
| torR_check_R | ACCAGAATAATCCCCACCGTTGAGC |
| envZ_K434T | TGCAAGAGATCTACAAGATTCTGGTGGTCG |
| envZ_check_R | ATCTCCCGACGGAAAGCGGGAGGCA |
| phoR_K031S | GGCTTAGATCTGGTCGAAGCGGAAGATTAT |
| phoR_check_R | TTTCGCAGCTGCATCTGGTGTAAGC |
| phoQ_check_R | TTCGATTTCAGCCAGTCAGGCTGGA |
| For luciferase reporter plasmid | |
| mgtA_Lux_F | TCGTCTTCACCTCGACTACGCCGTCGATATTACGCCGTTT |
| mgtA_Lux_R | ACTAACTAGAGGATCAAGGAGTCCCTCCGCACTGTCTGAA |
| pstS_Lux_F | TCGTCTTCACCTCGATGTGGAAGAGGTGATTGCACCGATC |
| pstS_Lux_R | ACTAACTAGAGGATCAATGTCTCCTGGGAGGATTCATAAA |
| ompC_Lux_F | TCGTCTTCACCTCGAAAACAAAGATTGCTGGAAATTATGC |
| ompC_Lux_R | ACTAACTAGAGGATCCCTGCTACCAGCAGAGCTGGGACCA |
| pLux_R | CCGTCCATTTGTGATAATAGTGG |
| For RR expression plasmid | |
| PHOPF-1 | TAGCGAATTCGAGCTAGGAGGAATTCACCATGCGCGTACTGGTTGTTGA |
| PHOPR-1 | CAAAACAGCCAAGCTTTACTATTTATCGTCGTCATCTTTGTAGTCGCGCAATTCGAACAGATAGC |
| PHOBF-1 | TAGCGAATTCGAGCTAGGAGGAATTCACCATGGCGAGACGTATTCTGGT |
| PHOBR-1 | CAAAACAGCCAAGCTTTACTATTTATCGTCGTCATCTTTGTAGTCAAAGCGGCTTGAAAAACGAT |
| OMPRF-1 | TAGCGAATTCGAGCTAGGAGGAATTCACCATGCAAGAGAACTACAAGATTCT |
| OMPRR-1 | CAAAACAGCCAAGCTTTACTATTTATCGTCGTCATCTTTGTAGTCTGCTTTAGAGCCGTCCGGTA |

Table S4. The amino acid sequence of OmpR family RRs.

| Sub-family | Bactaral strain | Gene name | Amino acid sequence |
|---|---|---|---|
| A | *Escherichia coli* str. K-12 substr. MG1655 | *arcA* | MQTPHILIVEDELVTRNTLKSIFEAEGYDVFEATDGAEMHQILSEYDINLVIMDINLPGKNGLLLARELREQANVALMFLTGRDNEVDKILGLEIGADDYITKPFNPRELTIRARNLLSRTMNLGTVSEERRSVESYKFNGWELDINSRSLIGPDGEQYKLPRSEFRAMLHFCENPGKIQSRAELLKKMTGRELKPHDRTVDVTIRRIRKHFESTPDTPEIIATIHGEGYRFCGDLED |
| A | *Escherichia coli* str. K-12 substr. MG1655 | *ompR* | MQENYKILVVDDDMRLRALLERYLTEQGFQVRSVANAEQMDRLLTRESFHLMVLDLMLPGEDGLSICRRLRSQSNPMPIIMVTAKGEEVDRIVGLEIGADDYIPKPFNPRELLARIRAVLRRQANELPGAPSQEEAVIAFGKFKLNLGTREMFREDEPMPLTSGEFAVLKALVSHPREPLSRDKLMNLARGREYSAMERSIDVQISRLRRMVEEDPAHPRYIQTVWGLGYVFVPDGSKA |
| A | *Escherichia coli* str. K-12 substr. MG1655 | *rstA* | MNTIVFVEDDAEVGSLIAAYLAKHDMQVTVEPRGDQAEETILRENPDLVLLDIMLPGKDGMTICRDLRAKWSGPIVLLTSLDSDMNHILALEMGACDYILKTTPPAVLLARLRLHLRQNEQATLTKGLQETSLTPYKALHFGTLTIDPINRVVTLANTEISLSTADFELLWELATHAGQIMDRDALLKNLRGVSYDGLDRSVDVAISRLRKKLLDNAAEPYRIKTVRNKGYLFAPHAWE |
| A | *Escherichia coli* str. K-12 substr. MG1655 | *torR* | MPHHIVIVEDEPVTQARLQSYFTQEGYTVSVTASGAGLREIMQNQSVDLILLDINLPDENGLMLTRALRERSTVGIILVTGRSDRIDRIVGLEMGADDYVTKPLELRELVVRVKNLLWRIDLARQAQPHTQDNCYRFAGYCLNVSRHTLERDGEPIKLTRAEYEMLVAFVTNPGEILSRERLLRMLSARRVENPDLRTVDVLIRRLRHKLSADLLVTQHGEGYFLAADVC |
| A | *Pseudomonas aeruginosa* PAO1 | *parR* | MDCPTLSKVLLVEDDQKLARLIASFLSQHGFEVRQVHRGDAAFAAFLDFKPQVVVLDLMLPGQNGLQVCREIRRVANLPILILTAQEDDLDHILGLESGADDYVIKPIEPPVLLARLRALMRRHAPLPASPESLTFGKLNIDRRRREAELEGLGIELTTMEFELLWLLASQAGEILSRDEILNQIRGIGFDGLNRSVDVCISKLRNKLKDNPREPVRIKTVWGKGYLFNPLGWEL |
| A | *Pseudomonas aeruginosa* PAO1 | *gltR* | MSANGRSILLVDDDQEIRELLETYLSRAGFQVRSVSRGADFRQALCEEEASLAILDVMLPDEDGFSLCRWIRSHQRLACMPIIMLTASSDEADRVIGLELGADDYLGKPFSPRELLARIKALLRRAQFTQVRGGDVLAFEDWRLDTVSHRLFHEDGEEFFLSGADFALLKLFLDHPQQILDRDTIANATRGREVLPLERIVDMAVSRLRQRLRDTGKAPRLIQTVRGSGYLLAAQVRPHLQP |
| A | *Pseudomonas aeruginosa* PAO1 | *bfmR* | MEHVDHILIVDDDREIRELVGNYLKKNGLRTTIVADGRQMRAFLEANTVDLIVLDIMMPGDDGLLLCRELRVGKHKATPVLMLTARNDETDRIIGLEMGADDYLTKPFSARELLARINAVLRRTRMLPPNLTVSESSRLIGFGQWQLDTSARHLLDDAGTVVALSGAEYRLLRVFLDHPQRVLSRDQLLNLTQGREADIFDRSIDLLVSRLRQRLGDDAREPEYIKTVRSEGYVFSL |

| | | | |
|---|---|---|---|
| | | | PVRLVEAHP |
| A | *Pseudomonas aeruginosa* PAO1 | *PA4983* | MAMVPRVLVVDDDPVIRELLQAYLGEEGYDVLCAGNAEQAEACLAECAHLGQPVELVLLDIRLPGKDGLTLTRELRV RSEVGIILITGRNDEIDRIVGLECGADDYVIKPLNPRELVSRAKNLIRRVRHAQASAGPARQALRQFGDWLLDADRRRLI DHAGNETLLTHGEFQLLGAFLRNSGHTLSRDQLMDQIRNREWLPSDRSIDVLVGRLRRKLRDDPAEPQLIITIHGAGYL FTAAASDA |
| A | *Pseudomonas aeruginosa* PAO1 | *PA1157* | MEQEAWRILIVEDDRRLAELTREYLEGNGLKVDIEANGALAAARILAERPDLVVLDLMLPGEDGLSICRQVRPQFDGPI LMLTARTDDMDEVLGLEMGADDYVCKPVRPRVLLARIRALLRRSEAPEAGAPAADSKRLAFGRLVIDNAMREAWLD GTTIELTSAEFDLLWLLAANAGRILSREEIFNALRGIEYDGQDRSIDVRISRIRPKIGDDPMHPRLIKTVRSKGYLFVGEG |
| A | *Pseudomonas aeruginosa* PAO1 | *amgR* | MSNPAALAEGEKILVVDDDARLRRLLERFLDEQGYRVRAVENTEQMDRLLSRELFQLVVLDLMLPGEDGLTACRRLRE QNNQVPIIMLTAKGDEGSRIQGLELGADDYLAKPFNPRELLARIKAVLRRQAPLVPGAPAGADEVVTFGDYQLFLATRE LKKGDEVHMLTTGEFAVLKALVQHAREPLTRDKLMNLARGREWDALERSIDVQISRLRRLIEPDPSKPRYIQTVWGVG YVFVPDGNARKA |
| A | *Haemophilus influenzae* Rd KW20 | *arcA* | MTTPKILVVEDEIVTRNTLKGIFEAEGYDVFEAENGVEMHHILANHNINLVVMDINLPGKNGLLLARELREELSLPLIFL TGRDNEVDKILGLEIGADDYLTKPFNPRELTIRARNLLHRAMPHQEKENTFGREFYRFNGWKLDLNSHSLITPEGQEFK LPRSEFRAMLHFCENPGKLQTREELLKKMTGRELKPQDRTVDVTIRRIRKHFEDHPNTPNIIMTIHGEGYRFCGDIE |
| A | *Salmonella enterica* subsp. Enterica serovar Typhimurium str. LT2 | *rstA* | MNRIVFVEDDAEVGSLIAAYLAKHDIDVIVEPRGDRAEDLILTTQPDLVLLDIMLPGKDGMTICRDLRHRWQGPIVLLTS LDSDMNHILALEMGACDYILKTTPPAVLLARLRLHLRQSEQTQQAKSLQESALTPHKALRFGALTIDPLNRAVQLNGDF ISLSTADFELLWELATHAGQIMDRDALLKTLRGVNYDGLDRSVDVAISRLRKKLLDSAAEPYRIKTIRNKGYLFAPHAW DETTG |
| A | *Salmonella enterica* subsp. Enterica serovar Typhimurium str. LT2 | *ompR* | MQENYKILVVDDDMRLRALLERYLTEQGFQVRSVANAEQMDRLLTRESFHLMVLDLMLPGEDGLSICRRLRSQSNPM PIIMVTAKGEEVDRIVGLEIGADDYIPKPFNPRELLARIRAVLRRQANELPGAPSQEEAVIAFGKFKLNLGTREMFREDEP MPLTSGEFAVLKALVSHPREPLSRDKLMNLARGREYSAMERSIDVQISRLRRMVEEDPAHPRYIQTVWGLGYVFVPDG SKA |
| A | *Salmonella enterica* subsp. Enterica serovar Typhimurium | *torR* | MSHHIVIVEDEPVTQARLQAYFEQEGYRVSVTDSGAGLRDIMEHEHVSLILLDINLPDENGLMLTRALRERSTVGIILVT GRCDQIDRIVGLEMGADDYVTKPLELRELVVRVKNLLWRIDLARPTPQNASENCYMFSGYCLNVMNHTLEHNGEAIK LTRAEYELLLAFVTNPGKVLHRERLLRMLSARRVETPDLRTIDVLVRRLRHKITPELLVTQHGEGYFLASEVY |

| | | | |
|---|---|---|---|
| | str. LT2 | | |
| A | *Salmonella enterica* subsp. Enterica serovar Typhimurium str. LT2 | *arcA* | MQTPHILIVEDELVTRNTLKSIFEAEGYDVFEATDGAEMHQILSEYDINLVIMDINLPGKNGLLLARELREQANVALMFLTGRDNEVDKILGLEIGADDYITKPFNPRELTIRARNLLSRTMNLGTVSEERRSVESYKFNGWELDINSRSLIGPDGEQYKLPRSEFRAMLHFCENPGKIQSRAELLKKMTGRELKPHDRTVDVTIRRIRKHFESTPDTPEIIATIHGEGYRFCGDLQD |
| A | *Shigella dysenteriae* 1617 | *Asd161705396* | MKPVVLVVDDDTAICALLQDVLSEHVFTVSVCHTGQEAILRIEGDPDIALVVLDMMLPDTNGLRVLQQIQKLRPTLPVVMLTGMGSKSDVVVGLEMGADDYICKPFTPRVVVARLKAVLRRVGALAVNDEKSAGLSFNGWYLDTMRCQLHNPLQQHVELTQGEYGLLLALAQNASRVLSREQLLAFTHSDSVEVFDRTIDVLIMRLRRKIELNPHQPMLIKTLRGLGYVFAADVH |
| A | *Shigella dysenteriae* 1617 | *rstA* | MTFFISTVNVMNTIVFVEDDAEVGSLIAAYLAKHDMQVTVEPRGDQAEETILRENPDLVLLDIMLPGKDGMTICRDLRAKWSGPIVLLTSLDSDMNHILALEMGACDYMPPAVLLARLRLHLRQNEQATLTKGLQETSLTPYKALHFGTLTIDPINRVVTLANTEISLSTADFELLWELATHAGQIMDRDALLKNLRGVSYDGLDRSVDVAISRLRKKLLDNAAEPYRIKTVRNKGYLFAPHAWE |
| A | *Shigella dysenteriae* 1617 | *ompR* | MGVQTMQENYKILVVDDDMRLRALLERYLTEQGFQVRSVANAEQMDRLLTRESFHLMVLDLMLPGEDGLSICRRLRSQSNPMPIIMVTAKGEEVDRIVGLEIGADDYIPKPFNPRELLARIRAVLRRQANELPGAPSQEEAVIAFGKFKLNLGTREMFREDEPMPLTSGEFAVLKALVSHPREPLSRDKLMNLARGREYSAMERSIDVQISRLRRMVEEDPAHPRYIQTVWGLGYVFVPDGSKA |
| B | *Escherichia coli* str. K-12 substr. MG1655 | *baeR* | MTELPIDENTPRILIVEDEPKLGQLLIDYLRAASYAPTLISHGDQVLPYVRQTPPDLILLDLMLPGTDGLTLCREIRRFSDIPIVMVTAKIEEIDRLLGLEIGADDYICKPYSPREVVARVKTILRRCKPQRELQQQDAESPLIIDEGRFQASWRGKMLDLTPAEFRLLKTLSHEPGKVFSREQLLNHLYDDYRVVTDRTIDSHIKNLRRKLESLDAEQSFIRAVYGVGYRWEADACRIV |
| B | *Escherichia coli* str. K-12 substr. MG1655 | *creB* | MQRETVWLVEDEQGIADTLVYMLQQEGFAVEVFERGLPVLDKARKQVPDVMILDVGLPDISGFELCRQLLALHPALPVLFLTARSEEVDRLLGLEIGADDYVAKPFSPREVCARVRTLLRRVKKFSTPSPVIRIGHFELNEPAAQISWFDTPLALTRYEFLLLKTLLKSPGRVWSRQQLMDSVWEDAQDTYDRTVDTHIKTLRAKLRAINPDLSPINTHRGMGYSLRGL |
| B | *Escherichia coli* str. K-12 substr. MG1655 | *phoB* | MARRILVVEDEAPIREMVCFVLEQNGFQPVEAEDYDSAVNQLNEPWPDLILLDWMLPGGSGIQFIKHLKRESMTRDIPVVMLTARGEEEDRVRGLETGADDYITKPFSPKELVARIKAVMRRISPMAVEEVIEMQGLSLDPTSHRVMAGEEPLEMGPTEFKLLHFFMTHPERVYSREQLLNHVWGTNVYVEDRTVDVHIRRLRKALEPGGHDRMVQTVRGTGYRFSTRF |

| | | | |
|---|---|---|---|
| B | *Bacillus subtilis* substr. subtilis str. 168 | *yclK* | MKILMIEDNVSVCTMTEMFFFKEGFEAEFVHDGLEGYQRFTEENWDLIILDIMLPSMDGVTICRKIRETSTVPIIMLTAKDTESDQVIGFEMGADDYVTKPFSPLTLVARIKAVIRRYKATGKAVIDEDMIETECFTINKKTREVLLNGEPVENLTPKEFDLLYYLVQNPRQVFSREQLLEQVWGYQFYGDERTVDVHIKRLRKKLASEDKPFLYTVWGVGYKFDED |
| B | *Bacillus subtilis* substr. subtilis str. 168 | *yycF* | MDKKILVVDDEKPIADILEFNLRKEGYEVHCAHDGNEAVEMVEELQPDLILLDIMLPNKDGVEVCREVRKKYDMPIIMLTAKDSEIDKVIGLEIGADDYVTKPFSTRELLARVKANLRRQLTTAPAEEEPSSNEIHIGSLVIFPDAYVVSKRDETIELTHREFELLHYLAKHIGQVMTREHLLQTVWGYDYFGDVRTVDVTVRRLREKIEDNPSHPNWIVTRRGVGYYLRNPEQD |
| B | *Bacillus subtilis* substr. subtilis str. 168 | *resD* | MDQTNETKILVVDDEARIRRLLRMYLERENYAIDEAENGDEAIAKGLEANYDLILLDLMMPGTDGIEVCRQIREKKATPIIMLTAKGEEANRVQGFEAGTDDYIVKPFSPREVVLRVKALLRRASQTSYFNANTPTKNVLVFSHLSIDHDAHRVTADGTEVSLTPKEYELLYFLAKTPDKVYDREKLLKEVWQYEFFGDLRTVDTHVKRLREKLNKVSPEAAKKIVTVWGVGYKFEVGAE |
| B | *Bacillus subtilis* substr. subtilis str. 168 | *phoP* | MNKKILVVDDEESIVTLLQYNLERSGYDVITASDGEEALKKAETEKPDLIVLDVMLPKLDGIEVCKQLRQQKLMFPILMLTAKDEEFDKVLGLELGADDYMTKPFSPREVNARVKAILRRSEIAAPSSEMKNDEMEGQIVIGDLKILPDHYEAYFKESQLELTPKEFELLLYLGRHKGRVLTRDLLLSAVWNYDFAGDTRIVDVHISHLRDKIENNTKKPIYIKTIRGLGYKLEEPKMNE |
| B | *Mycobacterium tuberculosis* H37Rv | *regX* | MTSVLIVEDEESLADPLAFLLRKEGFEATVVTDGPAALAEFDRAGADIVLLDLMLPGMSGTDVCKQLRARSSVPVIMVTARDSEIDKVVGLELGADDYVTKPYSARELIARIRAVLRRGGDDDSEMSDGVLESGPVRMDVERHVVSVNGDTITLPLKEFDLLEYLMRNSGRVLTRGQLIDRVWGADYVGDTKTLDVHVKRLRSKIEADPANPVHLVTVRGLGYKLEG |
| B | *Mycobacterium tuberculosis* H37Rv | *Rv0818* | MLELLLLTSELYPDPVLPALSLLPHTVRTAPAEASSLLEAGNADAVLVDARNDLSSGRGLCRLLSSTGRSIPVLAVVSEGGLVAVSADWGLDEILLLSTGPAEIDARLRLVVGRRGDLADQESLGKVSLGELVIDEGTYTARLRGRPLDLTYKEFELLKYLAQHAGRVFTRAQLLHEVWGYDFFGGTRTVDVHVRRLRAKLGPEHEALIGTVRNVGYKAVRPARGRPPAADPDDEDADPGRDGMQEPLVDPLRSQ |
| B | *Mycobacterium tuberculosis* H37Rv | *Rv2884* | MPTGPTTGKWHPHEVWRYLLEVLLLTDEADLESALPELESFAQSVQRAPLDDPGAAKGADADVAIIDARADLAAARRVCRRLTTSAPALAVVAVVAPANFVAVDGDWIFDDVLLNAAGGAELQARLRLAITRRRSTLAGTLQFGDLVLHPASYTASLGDRDLGLTLTEFKLMNFLVQHAGRAFTRTRLMREVWGYECHGRIRTVDVHVRRLRAKLGAEHESMIDTVRGVGYMAVTPPQPRWIISESILNRCK |
| B | *Mycobacterium* | *mtrA* | MDTMRQRILVVDDDASLAEMLTIVLRGEGFDTAVIGDGTQALTAVRELRPDLVLLDLMLPGMNGIDVCRVLRADSGVP |

| | | | |
|---|---|---|---|
| | *tuberculosis* H37Rv | | IVMLTAKTDTVDVVLGLESGADDYIMKPFKPKELVARVRARLRRNDDEPAEMLSIADVEIDVPAHKVTRNGEQISLTPLEFDLLVALARKPRQVFTRDVLLEQVWGYRHPADTRLVNVHVQRLRAKVEKDPENPTVVLTVRGVGYKAGPP |
| B | *Pseudomonas aeruginosa* PAO1 | *creB* | MPHILIVEDEAAIADTLLYALQAEGFATTWVTLAGEALALQERQPADLLILDVGLPDISGFEACKRLRRFSEVPVIFLTARDAEIDRVVGLEIGADDYVVKPFSPREVAARVKAILKRMAPRPAALEEAAPSGPFQVDEERVRIHYRDTPLNLTRHEFRLLQTLLGQPERVFSREQLLDALGVASEAGYERNIDSHIKSLRAKLRQVNERGEAIQTHRGLGYSYSPDHA |
| B | *Pseudomonas aeruginosa* PAO1 | *phoB* | MVGKTILIVDDEAPIREMIAVALEMAGYECLEAENTQQAHAVIVDRKPDLILLDWMLPGTSGIELARRLKRDELTVDIPIIMLTAKGEEDNKIQGLEVGADDYITKPFSPRELVARLKAVLRRTGPGDSEAPIEVGGLLLDPISHRVTIDGKPAEMGPTEYRLLQFFMTHQERAYTRGQLLDQVWGGNVYVEERTVDVHIRRLRKALGEVYENLVQTVRGTGYRFSTKS |
| B | *Corynebacterium glutamicum* ATCC 13032 | *cgtR4* | MTRILIVEDEESLADPLAFLLRKEGFDTIIAGDGPTALVEFSRNEIDIVLLDLMLPGMSGTDVCKELRSVSTVPVIMVTARDSEIDKVVGLELGADDYVTKPYSSRELIARIRAVLRRRGVTETEAEELPLDDQILEGGRVRMDVDSHTVTVGGEPVSMPLKEFDLLEYLLRNAGRVLTRGQLIDRIWGADYVGDTKTLDVHVKRLRSKIEEEPSRPRYLVTVRGLGYKFEL |
| B | *Corynebacterium glutamicum* ATCC 13032 | *mtrA* | MSQKILVVDDDPAISEMLTIVLSAEGFDTVAVTDGALAVETASREQPDLILLDLMLPGMNGIDICRLIRQESSVPIIMLTAKTDTVDVVLGLESGADDYVNKPFKAKELVARIRARLRATVDEPSEIIEVGDLSIDVPAHTVKRNGAEISLTPLEFDLLLELARKPQQVFTREELLGKVWGYRHASDTRLVNVHVQRLRAKIEKDPENPQIVLTVRGVGYKTGHND |
| B | *Corynebacterium glutamicum* ATCC 13032 | *cgtR5* | MTNPSPALNETLSGRVLIVEDERPLARMISLYLSKAGFDTTTIHDGAAAPDKVAHLRPDVVILDLGLPGLDGLEVCKRIRAFTDCYILMLTARGSERDRITGLEIGADDYITKPFNIRELVIRIQSVMRRPRKIDETIQNGLTLTYGHIELDTLAHEVTVKGVGVTLTRTEFELLQALMHKPGEAVSRRDLVSQVWDTTWVGDERIVDVHIGNLRRKLEAPAPGSHFIDTIRGVGYRMAFK |
| B | *Corynebacterium glutamicum* ATCC 13032 | *cgtR9* | MADRTPTTATPPGRVLVVDDEQPLAQMVASYLIRAGFDTRQAHTGTQAVDEARRFSPDVVVLDLGLPELDGLEVCRRIRTFSDCYILMLTARGSEDDKISGLTLGADDYITKPFSIRELVTRVHAVLRRPRTSTTPPQVTTPLIVGDLILDPVAHQVWVGETTVELTRTEFELLVALALRPGQVLTRHDLITEVWDTTWVGDERIVDVHIGNLRRKLGTDTRGRGFIDTVRGVGYRVGQP |
| B | *Haemophilus influenzae* Rd KW20 | *phoB* | MTRKILIVEDECAIREMIALFLSQKYYDVIEASDFKTAINKIKENPKLILLDWMLPGRSGIQFIQYIKKQESYAAIPIIMLTAKSTEEDCIACLNAGADDYITKPFSPQILLARIEAVWRRIYEQQSQFIQIDELSIDENAQRVFFQQQEINLSSTEFKLLHFFMRHPEKVYSREQLLNRIWHNDLEVEYRTVDSYIRRLRRNLAPFQCEHYIQTVRGSGYRFSSYLRDKQ |
| B | *Salmonella enterica* | *phoB* | MARRILVVEDEAPIREMVCFVLEQNGFQPVEAEDYDSAVNKLNEPWPDLILLDWMLPGGSGLQFIKHLKREAMTRDIP |

| | | | |
|---|---|---|---|
| | subsp. Enterica serovar Typhimurium str. LT2 | | VVMLTARGEEEDRVRGLETGADDYITKPFSPKELVARIKAVMRRISPMAVEEVIEMQGLSLDPGSHRVMTGDSPLDMGPTEFKLLHFFMTHPERVYSREQLLNHVWGTNVYVEDRTVDVHIRRLRKALEHSGHDRMVQTVRGTGYRFSTRF |
| B | *Salmonella enterica* subsp. Enterica serovar Typhimurium str. LT2 | *baeR* | MTELPIDENTPRILIVEDEPKLGQLLIDYLRAASYAPTLINHGDKVLPYVRQTPPDLILLDLMLPGTDGLTLCREIRRFSDIPIVMVTAKIEEIDRLLGLEIGADDYICKPYSPREVVARVKTILRRCKPQRELQQQDAESPLMIDESRFQASWCGKALDLTPAEFRLLKTLSLEPGKVFSREQLLNHLYDDYRVVTDRTIDSHIKNLRRKLESLDAEQSFIRAVYGVGYRWEADACRLV |
| B | *Salmonella enterica* subsp. Enterica serovar Typhimurium str. LT2 | *creB* | MQQPQVWLVEDEQGIADTLIYTLQLEGFTVELFARGLPALEKARQQRPDAVILDVGLPDISGFELCRQLLERHPALPILFLTARSDEVDRLLGLEIGADDYVAKPFSPREVSARVRTLLRRVKKFAAPSPVVRTGHFELNEPAAQIAWFGTPLSLTRYEFLLLKTLLLSPERVYSRQQLMDIVWSDAQETFDRTVDTHIKTLRAKLRAINPELSPINTHRGMGYSLRSV |
| B | *Shigella dysenteriae* 1617 | *phoB* | MARRILVVEDEAPIREMVCFVLEQNGFQPVEAEDYDSAVNQLNEPWPDLILLDWMLPGGSGIQFIKHLKRESMTRDIPVVMLTARGEEEDRVRGLETGADDYITKPFSPKELVARIKAVMRRISPMAVEEVIEMQGLSLDPTSHRVMTGEEPLEMGPTEFKLLHFFMTHPERVYSREQLLNHVWGTNVYVEDRTVDVHIRRLRKALEPGGHDRMVQTVRGTGYRFSTRF |
| B | *Shigella dysenteriae* 1617 | *baeR* | MTELPIDENTPRILIVEDEPKLGQLLIDYLRAASYAPTLISHGDQVLPYVRQTPPDLILLDLMLPGTDGLTLCREIRRFSDIPIVMVTAKIEEIDRLLGLEIGADDYICKPYSPREVVARVKTILRRCKPQRELQQQDAESPLIIDEGRFQASWRGKMLDLTPAEFRLLKTLSHEPGKVFSREQLLNHLYDDYRVVTDRTIDSHIKNLRRKLESLDAEQSFIRAVYGVGYRWEADACRIV |
| B | *Shigella dysenteriae* 1617 | *creB* | MQRETVWLVEDEQGIADTLVYMLQQEGFAVEVFERGLPVLDKARQQAPDVMILDVGLPDISGFELCRQLLALHPALPVLFLTARSEEVDRLLGLEIGADDYVAKPFSPREVCARVRTLLRRVKKFSSPSPVIRIGHFELNEPAAQISWFDTPLTLTRYEFLLLKTLLKSPGRVWSRQQLMDSVWEDAQDTYDRTVDTHIKTLRAKLRAINPDLSPINTHRGMGYSLRGL |
| C | *Escherichia coli* str. K-12 substr. MG1655 | *basR* | MKILIVEDDTLLLQGLILAAQTEGYACDSVTTARMAEQSLEAGHYSLVVLDLGLPDEDGLHFLARIRQKKYTLPVLILTARDTLTDKIAGLDVGADDYLVKPFALEELHARIRALLRRHNNQGESELIVGNLTLNMGRRQVWMGGEELILTPKEYALLSRLMLKAGSPVHREILYNDIYNWDNEPSTNTLEVHIHNLRDKVGKARIRTVRGFGYMLVANEEN |
| C | *Escherichia coli* str. K-12 substr. MG1655 | *cpxR* | MNKILLVDDDRELTSLLKELLEMEGFNVIVAHDGEQALDLLDDSIDLLLLDVMMPKKNGIDTLKALRQTHQTPVIMLTARGSELDRVLGLELGADDYLPKPFNDRELVARIRAILRRSHWSEQQQNNDNGSPTLEVDALVLNPGRQEASFDGQTLELTGTEFTLLYLLAQHLGQVVSREHLSQEVLGKRLTPFDRAIDMHISNLRRKLPDRKDGHPWFKTLRGRGYLMVSAS |

| | | | |
|---|---|---|---|
| C | *Escherichia coli* str. K-12 substr. MG1655 | *cusR* | MKLLIVEDEKKTGEYLTKGLTEAGFVVDLADNGLNGYHLAMTGDYDLIILDIMLPDVNGWDIVRMLRSANKGMPILLLTALGTIEHRVKGLELGADDYLVKPFAFAELLARVRTLLRRGAAVIIESQFQVADLMVDLVSRKVTRSGTRITLTSKEFTLLEFFLRHQGEVLPRSLIASQVWDMNFDSDTNAIDVAVKRLRGKIDNDFEPKLIQTVRGVGYMLEVPDGQ |
| C | *Escherichia coli* str. K-12 substr. MG1655 | *kdpE* | MTNVLIVEDEQAIRRFLRTALEGDGMRVFEAETLQRGLLEAATRKPDLIILDLGLPDGDGIEFIRDLRQWSAVPVIVLSARSEESDKIAALDAGADDYLSKPFGIGELQARLRVALRRHSATTAPDPLVKFSDVTVDLAARVIHRGEEEVHLTPIEFRLLAVLLNNAGKVLTQRQLLNQVWGPNAVEHSHYLRIYMGHLRQKLEQDPARPRHFITETGIGYRFML |
| C | *Escherichia coli* str. K-12 substr. MG1655 | *phoP* | MRVLVVEDNALLRHHLKVQIQDAGHQVDDAEDAKEADYYLNEHIPDIAIVDLGLPDEDGLSLIRRWRSNDVSLPILVLTARESWQDKVEVLSAGADDYVTKPFHIEEVMARMQALMRRNSGLASQVISLPPFQVDLSRRELSINDEVIKLTAFEYTIMETLIRNNGKVVSKDSLMLQLYPDAELRESHTIDVLMGRLRKKIQAQYPQEVITTVRGQGYLFELR |
| C | *Escherichia coli* str. K-12 substr. MG1655 | *qseB* | MRILLIEDDMLIGDGIKTGLSKMGFSVDWFTQGRQGKEALYSAPYDAVILDLTLPGMDGRDILREWREKGQREPVLILTARDALAERVEGLRLGADDYLCKPFALIEVAARLEALMRRTNGQASNELRHGNVMLDPGKRIATLAGEPLTLKPKEFALLELLMRNAGRVLSRKLIEEKLYTWDEEVTSNAVEVHVHHLRRKLGSDFIRTVHGIGYTLGEK |
| C | *Escherichia coli* str. K-12 substr. MG1655 | *hprR* | MKILLIEDNQRTQEWVTQGLSEAGYVIDAVSDGRDGLYLALKDDYALIILDIMLPGMDGWQILQTLRTAKQTPVICLTARDSVDDRVRGLDSGANDYLVKPFSFSELLARVRAQLRQHHALNSTLEISGLRMDSVSHSVSRDNISITLTRKEFQLLWLLASRAGEIIPRTVIASEIWGINFDSDTNTVDVAIRRLRAKVDDPFPEKLIATIRGMGYSFVAVKK |
| C | *Bacillus subtilis* substr. subtilis str. 168 | *ykoH* | MEKGHILIVEDEEKIARVLQLELEYEGYSVTIKHNGTEGLDAAAEGGYSLVLLDVMLPGLSGLEVLRRLRKTDSQTPVILLTARDSIPDKVTGLDIGANDYVTKPFEIEELLARIRAALRQNGTKTEDIGTFLTYDDLRVNEKTREVRRGDKEVELTPREFDLLVYMLKHPQQVLTREQILSSVWGFDYIGDTNVVDVYIRYIRKKLDYPYEKQLIHTIRGVGYAIKG |
| C | *Bacillus subtilis* substr. subtilis str. 168 | *cssR* | MSYTIYLVEDEDNLNELLTKYLENEGWNITSFTKGEDARKKMTPSPHLWILDIMLPDTDGYTLIKEIKAKDPDVPVIFISARDADIDRVLGLELGSNDYISKPFLPRELIIRVQKLLQLVYKEAPPVQKNEIAVSSYRVAEDAREVYDENGNIINLTSKEFDLLLLFIHHKGHPYSREDILLKVWGHDYFGTDRVVDDLVRRLRRKMPELKVETIYGFGYRMMSS |
| C | *Mycobacterium tuberculosis* H37Rv | *tcrA* | MADETTMRAGRGPGRACGRVSGVRILVVEDEPKMTALLARALTEEGHTVDTVADGRHAVAAVDGGDYDAVVLDVMLPGIDGFEVCARLRRQRVWTPVLMLTARGAVTDRIAGLDGGADDYLTKPFNLDELFARLRALSRRGPIPRPPTLEAGDLRLDPSEHRVWRADTEIRLSHKEFTLLEALIRRPGIVHTRAQLLERCWDAAYEARSNIVDVYIRYLRDKIDRPFGVTSLETIRGAGYRLRKDGGRHALPR |
| C | *Mycobacterium* | *phoP* | MRKGVDLVTAGTPGENTTPEARVLVVDDEANIVELLSVSLKFQGFEVYTATNGAQALDRARETRPDAVILDVMMPGM |

| | | | |
|---|---|---|---|
| | *tuberculosis* H37Rv | | DGFGVLRRLRADGIDAPALFLTARDSLQDKIAGLTLGGDDYVTKPFSLEEVVARLRVILRRAGKGNKEPRNVRLTFADIELDEETHEVWKAGQPVSLSPTEFTLLRYFVINAGTVLSKPKILDHVWRYDFGGDVNVVESYVSYLRRKIDTGEKRLLHTLRGVGYVLREPR |
| C | *Mycobacterium tuberculosis* H37Rv | *prrA* | MGGMDTGVTSPRVLVVDDDSDVLASLERGLRLSGFEVATAVDGAEALRSATENRPDAIVLDINMPVLDGVSVVTALRAMDNDVPVCVLSARSSVDDRVAGLEAGADDYLVKPFVLAELVARVKALLRRRGSTATSSSETITVGPLEVDIPGRRARVNGVDVDLTKREFDLLAVLAEHKTAVLSRAQLLELVWGYDFAADTNVVDVFIGYLRRKLEAGGGPRLLHTVRGVGFVLRMQ |
| C | *Mycobacterium tuberculosis* H37Rv | *kdpE* | MTLVLVIDDEPQILRALRINLTVRGYQVITASTGAGALRAAAEHPPDVVILDLGLPDMSGIDVLGGLRGWLTAPVIVLSARTDSSDKVQALDAGADDYVTKPFGMDEFLARLRAAVRRNTAAAELEQPVIETDSFTVDLAGKKVIKDGAEVHLTPTEWGMLEMLARNRGKLVGRGELLKEVWGPAYATETHYLRVYLAQLRRKLEDDPSHPKHLLTESGMGYRFEA |
| C | *Mycobacterium tuberculosis* H37Rv | *trcR* | MTTMSGYTRSQRPRQAILGQLPRIHRADGSPIRVLLVDDEPALTNLVKMALHYEGWDVEVAHDGQEAIAKFDKVGPDVLVLDIMLPDVDGLEILRRVRESDVYTPTLFLTARDSVMDRVTGLTSGADDYMTKPFSLEELVARLRGLLRRSSHLERPADEALRVGDLTLDGASREVTRDGTPISLSSTEFELLRFLMRNPRRALSRTEILDRVWNYDFAGRTSIVDLYISYLRKKIDSDREPMIHTVRGIGYMLRPPE |
| C | *Mycobacterium tuberculosis* H37Rv | *tcrX* | MRRADGQPVTVLVVDDEPVLAEMVSMALRYEGWNITTAGDGSSAIAAARRQRPDVVVLDVMLPDMSGLDVLHKLRSENPGLPVLLLTAKDAVEDRIAGLTAGGDDYVTKPFSIEEVVLRLRALLRRTGVTTVDSGAQLVVGDLVLDEDSHEVMRAGEPVSLTSTEFELLRFMMHNSKRVLSKAQILDRVWSYDFGGRSNIVELYISYLRKKIDNGREPMIHTLRGAGYVLKPAR |
| C | *Mycobacterium tuberculosis* H37Rv | *mrpA* | MRILVVDDDRAVRESLRRSLSFNGYSVELAHDGVEALDMIASDRPDALVLDVMMPRLDGLEVCRQLRGTGDDLPILVLTARDSVSERVAGLDAGADDYLPKPFALEELLARMRALLRRTKPEDAAESMAMRFSDLTLDPVTREVNRGQRRISLTRTEFALLEMLIANPRRVLTRSRILEEVWGFDFPTSGNALEVYVGYLRRKTEADGEPRLIHTVRGVGYVLRETPP |
| C | *Pseudomonas aeruginosa* PAO1 | *PA0756* | MRILLVEDHPQLAESVVQALKGAGWTVDLLQDGVAADLALASEEYALAILDVGLPRMDGFEVLARLRGRGKTLPVLMLTARGEVKDRVHGLNLGADDYLAKPFELSELEARVKALLRRSVLGGEQLQRCGALVYDLGTRRFSLDEQPLTLTSREQAVLEAMIARPGRVMSKEQLAAQVFGLDEEASADAIEIYVHRLRKKLEGGAVRIVTFRGLGYLLEAQGD |
| C | *Pseudomonas aeruginosa* PAO1 | *PA1437* | MRVLIVEDEAKTADYLNRGLSEQGFTVDLADNGIDGRHLALHGEYDVIVLDVMLPGVDGYGVLRALRERRQTPVIMLTARERVEDRVRGLREGADDYLIKPFSFLELVARLQALTRRGGNHESHSQMRIADLSIDLLSRKVFRGNTRLELTAKEYAL |

| | | | |
|---|---|---|---|
| | | | LCVLAQRSGEILSKTAIAELVWDINFDTDTNVVEVAIKRLRAKLDGPFENKLLHTIRGMGYVLENRALAESG |
| C | *Pseudomonas aeruginosa* PAO1 | *pfeR* | MIVQPSAVAKGWPIATTLLPASCGPADPKPEPKPKQRRLCRKVFPLRARCGALAHCPIPGKNEIFTNVNHSHISIPSPRLLLVEDDPRLREDLDAHFRRRGFRVTVCGDGSHGLEAAGREAFDLVLLDIMLPGLDGLALLESLRREQATPVMLMSALGAEQDRISGFTRGADDYLPKPFSLAELDARTDALLRRVRLDRLPLAQRRDTRLVFDDQAQDVLHQGLPAGLTPSEYRLLATLREHAGEALSKPFLYRSVLHRSYTRLDRGLDVHVCNLRRKLAVVAVRHLQIQAVRGQGYLLVETEHP |
| C | *Pseudomonas aeruginosa* PAO1 | *PA4032* | MRVAILDDESAELDRVEQTLQQIPAQGEQAWTVHRFARGEDLLKQLKRETFDLLILDWQLPDLSGLQLLRWSREHLDAPPPAIMLTSRDAEQDIVQALNSGADDYVSKPFRPNELKARVAAVLRRHGGTRPAQHEVQTFNDLSFDDAELTVTRAGAPISLTEREYRLARCLFANLGRPLSREYLYERFWPHEEVLSSRPLDTHIYRLRNKLGLTAERGWQLLTIYGYGYRLESVATVD |
| C | *Pseudomonas aeruginosa* PAO1 | *irlR* | MRILVIEDDTKTGEYLKKGLGESGYAVDWSQHGADGLYLALENRYDLVVLDVMLPGLDGWQIMEVLRKKHDVPVLFLTARDQLQDRIRGLELGADDYLVKPFSFTELLLRIRTLLRRGVVREAEQVQLADLQLDVLRRKVSRQGQVIALTNKEFALLHLLMRREGEVLSRTLIASEVWDMNFDSDTNVVDVAIKRLRAKVDNPFPNKLIHTVRGIGYVCEERPCPPAAP |
| C | *Pseudomonas aeruginosa* PAO1 | *PA0929* | MFPSLTPDPRLLAIEDDPTLGAHLFQHLNGSGFEVTWCRDGEEGLAAARSGGYDLILMDIMLPGRDGLEILRQLRQEQALPVILMSALGAEQDRIAGFSQGADDYLPKPFSLAELRVRIDAILRRIALERGGAPGRCAEPLARPSLQFSADVCDVSLGERFAGLTPTEYRLLETFLAAEGETLTKAFLYQHVLHRGHTQHDRSLDMHVSHLRRKLQRLGYAGHQLHTVWGKGYVLTPAP |
| C | *Pseudomonas aeruginosa* PAO1 | *PA2479* | MHVLLTEDDDLIASGIVAGLNAQGLTVDRVASAADTQALLQVARFDVLVLDLGLPDEDGLRLLQRLRQQGVDLPVLVLTARDAVTDRVAGLQAGADDYLLKPFDLRELGARLHTLQRRSAGRCVNVIEHGRLSYDPSTRETWLDGRPVELSRREQALLQALLNNRGRILSGEQLKDSVYGFGDEVESNALNVHIHHLRRKLGNAIVQTVRGLGYRLGPARGDGDDA |
| C | *Pseudomonas aeruginosa* PAO1 | *PA3204* | MSELLLIDDDRELCELLGTWLVQEGFSVRASHDGAQARRALAEQTPDAVVLDVMLPDGSGLELLKQLRGDHPDLPVLMLSARGEPLDRILGLELGADDYLAKPCDPRELTARLRAVLRRTHPAQPSAQMQLGDLSLNLTRGVAQIDGQEISLTLSESRILEALLRQPGEPLDKQALAQLALGRKLTLYDRSLDMHVSNLRKKLGSHPDGSPRILALRGRGYYYSH |
| C | *Pseudomonas aeruginosa* PAO1 | *PA2523* | MRILIIEDEVKTADYLHQGLTESGYIVDRANDGIDGLHMALQHPYELVILDVNLPGIDGWDLLRRLRERSSARVMMLTGHGRLTDKVRGLDLGADDFMVKPFQFPELLARVRSLLRRHDQAPMQDVLRVADLELDASRHRAFRGRVRINLTTKEFALLHLLMRRNGDVITRTQIISLIWDMNFDNDSNVVEVAICRLRAKIDDGFDLKLIHTIRGVGYVLEARR |
| C | *Pseudomonas* | *copR* | MKLLIVEDEPRIGQYLRQGLAEAGFAVDLSDDGNEGEQLALGGDYDLLILDVMLPGRDGWQILRSVRDAGMTVPVLF |

| | | | |
|---|---|---|---|
| | *aeruginosa* PAO1 | | LTARDAVEDRVRGLEQGADDYLVKPFAFVELLARVRTLLRRGSQQLQETTLQLADLELDLLRRRVQRQGKRIDLTAKEFALLELLLRRSGEVLPKSLIASQVWDMNFDSDTNVIEVAIRRLRAKVDDDYPQRLIHTVRGMGYVLEERDE |
| C | *Pseudomonas aeruginosa* PAO1 | *PA4381* | MRILVVEDNRDILANLADYLSLKGYTVDCAQDGLSGLHLAATEHYDLIVLDVMLPGIDGYALCRRLREDARRDTPVIMLTARDQLDDRLQGFRSGADDYLVKPFALSELSARIEAVLRRAQGGGRRELSVADLSYDLDTLEVKRAGKSLKLNPIGLKLLAVLMQKSPHVVRRDALEEAVWGDDCPDSDSLRSHVHQLRQVIDKPFSVALLHTVHGVGYRLAEEPNGV |
| C | *Pseudomonas aeruginosa* PAO1 | *phoP* | MKLLVVEDEALLRHHLYTRLGEQGHVVDAVPDAEEALYRVSEYHHDLAVIDLGLPGMSGLDLIRELRSQGKSFPILILTARGNWQDKVEGLAAGADDYVVKPFQFEELEARLNALLRRSSGFVQSTIEAGPLVLDLNRKQALVEEQPVALTAYEYRILEYLMRHHQQVVAKERLMEQLYPDDEERDANVIEVLVGRLRRKLEACGGFKPIDTVRGQGYLFTERCR |
| C | *Pseudomonas aeruginosa* PAO1 | *kdpE* | MTQLQNSILLIDDEPQIRKFLRISLNAQGYRVLEAGTGEEGLAQAALNRPDLVVLDLGLPDRDGQDILRDLREWSQVPVLVLSVRASEGEKVLALDGGANDYVTKPFGIQEFLARVRVLLRQAAQGESPEASVAVGPLQVDFAYRRVTLEGAEVALTRKEYAVLAALARHLGRVVTQQQLLKDIWGPTHVEDTHYLRVVVGHLRQKLGDDPASPRFLVTEAGVGYRLRDS |
| C | *Pseudomonas aeruginosa* PAO1 | *PA2657* | MRLLLVEDHVPLADELMASLTRQGYAVDWLADGRDAAVQGASEPYDLIILDLGLPGRPGLEILQEWRGLGLATPVLILTARGSWAERIDGLKAGADDYLTKPFHPEELALRIQALLRRAHGLANQSQLEAAGLRLDEQRQCVCLNGADVDLTAAEFRLLRYFMLHPGQVLSKGHLAEHLYDGETERDSNVIEVHVNHLRRKLGREVIETRRGQGYRYAGVAAG |
| C | *Pseudomonas aeruginosa* PAO1 | *PA3077* | MHIHVLVVEDNFDLAGTVIDYLEAAGVVCDHARDGQAGLNLARANRYDVILLDIMLPRINGRQVCRQLREAGLQTPVLMLTALDTLQDKLDGFDAGADDYLLKPFELPELLVRLQALSRRRSGQAQRLQVDDLVMDLDSRQASRGGTPLALSPTAWKILECLMRASPALVTREQLGRSVWGDEPPESNTLNVHMHHLRSTVDKGFATPLIHTLHSVGFQLERK |
| C | *Pseudomonas aeruginosa* PAO1 | *pmrA* | MRILLAEDDLLLGDGIRAGLRLEGDTVEWVTDGVAAENALVTDEFDLLVLDIGLPRRSGLDILRNLRHQGLLTPVLLLTARDKVADRVAGLDSGADDYLTKPFDLDELQARVRALTRRTTGRALPQLVHGELRLDPATHQVTLSGQAVELAPREYALLRLLLENSGKVLSRNQLEQSLYGWSGDVESNAIEVHVHHLRRKLGNQLIRTVRGIGYGIDQPAP |
| C | *Corynebacterium glutamicum* ATCC 13032 | *cgtR1* | MSKILLAEDDAGIADFIVRGLIREGFECEVTESGAEAFARAHSGDFDLMVLDLGLPHMDGTDVLEQLRNLQVTLPIIVLTARTNIEDRLRTLEGGADDYMPKPFQFAELLARIKLRLAKHTPQETPTDARVLRNGDLELDLRTQRVLIDGSWHDLSRREVDLLETLMRHPGQILSRVQLLRLVWDMDWDPGSNVVDVYIRALRKKIGAHRVETIRGSGYRLR |
| C | *Corynebacterium glutamicum* ATCC 13032 | *cgtR2* | MFQRVDVLIGRKSVTDWKTKRVSMKILVVDDEQAVRDSLRRSLSFNGYNVVLAEDGIQALEMIDKEQPALVILDVMMPGMDGLEVCRHLRSEGDDRPILILTARDNVSDRVGGLDAGADDYLAKPFALEELLARVRSLVRRSAVESNQSSSIEQALLSCGDLTLDPESRDVYRNGRAISLTRTEFALLQLLLKNQRKVLTRAQILEEVWGCDFPTSGNALEVYIGYLRRKTELEG |

| | | | |
|---|---|---|---|
| | | | EDRLIHTVRGVGYVLRETAP |
| C | *Corynebacterium glutamicum* ATCC 13032 | *cgtR3* | MDNQSDGQIRVLVVDDEPNIVELLTVSLKFQGFAVMTANDGNEALKIAREFRPDAYILDVMMPGMDGFELLTKLRGEGLDSPVLYLTAKDAVEHRIHGLTIGADDYVTKPFSLEEVITRLRVILRRGGAVEEDTSTSLQYADLTLNDETHEVTKAGELIDLSPTEFNLLRYLMLNAEVVLSKAKILDNVWHYDFGGDGNVVESYISYLRRKVDTQDPQLIQTVRGVGYVLRTPRS |
| C | *Haemophilus influenzae* Rd KW20 | *cpxR* | MSKLLLVDDDIELTELLSTLLELEGFDVETANNGLEALQKLNESYKLVLLDVMMPKLNGIETLKEIRKVSNVPVMMLTARGEDIDRVLGLELGADDCLPKPFNDRELIARIKAILRRSASPSNNISNVEILSFDGITLHFSHGIATYNEENLNLTDYEFKILCLLLKSKGNVVSREELSLEVMEKPLTPFDRSLDMHISNLRRKLPKRKNKPSWFKTLRGKGYALVT |
| C | *Haemophilus influenzae* Rd KW20 | *ygiX* | MRILLIEDDNLIGNGLQIGLTKLGFAVDWFTDGKTGMAALTSAPYDAVVLDLTLPKLDGLEVLQQWRSNHQDVPVLILTARDTLDERVKGLQSGADDYLCKPFALAEVAARLQALIRRRYGYHHSVIEQAGVKLDQNQRSVWLNNQPISLTSREYKLLELFMLNKDRVLSRSSIEEKLSSWDEEISSGALDVHIYNLRQKLGKQFIRTVHGVGYALGQVEK |
| C | *Salmonella enterica* subsp. Enterica serovar Typhimurium str. LT2 | *copR* | MTIMSSCWRFTDSLTSLWHTALMKILLIEDNQKTIEWVRQGLTEAGYVVDYACDGRDGLHLALQEHYSLIILDIMLPGLDGWQVLRALRTAHQSPVICLTARDSVEDRVKGLEAGANDYLVKPFSFAELLARVRAQLRQHVPAFTRLTINGLDMDATKQSVSRNGKPISLTRKEFLLLWLLASRAGEIVPRTAIASEVWGINFDSETNTVDVAIRRLRAKVDDPFEKKLIMTVRGMGYRLQAETSQNG |
| C | *Salmonella enterica* subsp. Enterica serovar Typhimurium str. LT2 | *kdpE* | MTNVLIVEDEQAIRRFLRAALEGDGLRVYEAETLQRGLLEAATRKPDLIILDLGLPDGDGIDFIRDLRQWSAIPVIVLSARSEESDKIAALDAGADDYLSKPFGIGELQARLRVALRRHAASPCADPIVRFSGVTVDLAARLIHRGDEEIHLTPIEFRLLAVLLNNTGKVLTQRQLLNQVWGPNAVEHSHYLRIYMGHLRQKLEQDPTRPRHFITETGIGYRFMP |
| C | *Salmonella enterica* subsp. Enterica serovar Typhimurium str. LT2 | *phoP* | MMRVLVVEDNALLRHHLKVQLQDSGHQVDAAEDAREADYYLNEHLPDIAIVDLGLPDEDGLSLIRRWRSSDVSLPVLVLTAREGWQDKVEVLSSGADDYVTKPFHIEEVMARMQALMRRNSGLASQVINIPPFQVDLSRRELSVNEEVIKLTAFEYTIMETLIRNNGKVVSKDSLMLQLYPDAELRESHTIDVLMGRLRKKIQAQYPHDVITTVRGQGYLFELR |
| C | *Salmonella enterica* subsp. Enterica serovar Typhimurium str. LT2 | *qseB* | MRILLVEDDTLIGDGIKAGLSKMGFSVDWFTEGRPGKEALYSAPYDAVILDLTLPGMDGRDILREWREKGKQEPVLILTARDALAERVEGLRLGADDYLCKPFALIEVAARLEALVRRASGQASSELRHGQVTLNPGNLVATLAGEPLALKPKEFALLELLLRNKGRVLPRKLIEEKLYNWDDDVSSNAVEVHVHHLRRKLGSEFIRTVHGIGYTLGDA |

| | | | |
|---|---|---|---|
| C | *Salmonella enterica* subsp. Enterica serovar Typhimurium str. LT2 | *cpxR* | MNKILLVDDDRELTSLLKELLEMEGFNVLVAHDGEQALELLDDSIDLLLLDVMMPKKNGIDTLKALRQTHQTPVIMLTARGSELDRVLGLELGADDYLPKPFNDRELVARIRAILRRSHWSEQQQSSDNGSPTLEVDALSLNPGRQEASFDGQTLELTGTEFTLLYLLAQHLGQVVSREHLSQEVLGKRLTPFDRAIDMHISNLRRKLPERKDGHPWFKTLRGRGYLMVSAS |
| C | *Salmonella enterica* subsp. Enterica serovar Typhimurium str. LT2 | *basR* | MKILIVEDDTLLLQGLILAAQTEGYACDGVSTARAAEHSLESGHYSLMVLDLGLPDEDGLHFLTRIRQKKYTLPVLILTARDTLNDRITGLDVGADDYLVKPFALEELHARIRALLRRHNNQGESELTVGNLTLNIGRHQAWRDGQELTLTPKEYALLSRLMLKAGSPVHREILYNDIYNWDNEPSTNTLEVHIHNLRDKVGKSRIRTVRGFGYMLVATEES |
| C | *Shigella dysenteriae* 1617 | *cpxR* | MNKILLVDDDRELTSLLKELLEMEGFNVIVAHDGEQALDLLDDSIDLLLLDVMMPKKNGIDTLKALRQTHQTPVIMLTARGSELDRVLGLELGADDYLPKPFNDRELVARIRAILRRSHWSEQQQNNDNGSPTLEVDALVLNPGRQEASFDGQTLELTGTEFTLLYLLAQHLGQVVSREHLSQEVLGKRLTPFDRAIDMHISNLRRKLPDRKDGHPWFKTLRGRGYLMVSAS |
| C | *Shigella dysenteriae* 1617 | *kdpE* | MTNVLIVEDEQAIRRFLRTALEGDGMRVFEAETLQRGLLEAATRKPDLIILDLGLPDGDGIEFIRDLRQWSAVPVIVLSARSEESDKIAALDAGADDYLSKPFGIGELQARMRVALRRHSATAAPDPLVKFSDVTVDLAARVIHRGEEEVHLTPIEFRLLAVLLNNAEKVLTQRQLLNQVWGPNAVEHSHYLRIYMGHLRQKLEQDPARPSHFITETGIGYRFML |
| C | *Shigella dysenteriae* 1617 | *qseB* | MRILLIEDDMLIGDGIKTGLSKMGFSVDWFTQGRQGKEALYSAPYDAVILDLTLPGMDGRDILREWREKGQREPVLILTARDALEERVEGLRLGADDYLCKPFALIEVAARLEALMRRTNGQTSNELRHGNVMLDPGKRIATLAGEPLTLKPKEFALLELLMRNAGRVLPRKLIEEKLYTWDEEVTSNAVEVHVHHLRRKLGSDFIRTVHGIGYTLGEK |
| C | *Shigella dysenteriae* 1617 | *yedW* | MSFEDSEKTSRVTLQQHYNFVMNQAVSITYDLWHIIFMKILLIEDNQRTQEWVTQGLSEAGYVIDAVSDGRDGLYLALKDDYALIILDIMLPGMDGWQILQTLRTAKQTPVICLTARDSVDDRVRGLDSGANDYLVKPFSFSELLARVRAQLRQHHALNSTLEISGLRMDSVSQSVSRDNISITLTRKEFQLLWLLASRAGEIIPRTVIASEIWGINFDSDTNTVDVAIRRLRAKVDDPFPVMLPISRTCVFQ |
| C | *Shigella dysenteriae* 1617 | *phoP* | MRVLVVEDNALLRHHLKVQIQDAGHQVDDAEDAKEADYYLNEHLPDIAIVDLGLPDEDGLSLIRRWRSNDVSLPILVLTARESWQDKVEVLSAGADDYVTKPFHIEEVMARMQALMRRNSGLASQVISLPPFQVDLSRRELSINDEVIKLTAFEYTIMETLIRNNGKVVSKDSLMLQLYPDAELRESHTIDVLMGRLRKKIQAQYPQEVITTVRGQGYLFELR |
| D | *Bacillus subtilis* substr. subtilis str. 168 | *ybdK* | MKGYRILIVEDDVMIGDLLQKILQREGYRVIWKTDGADVLSVIQKVDLVIMDVMLPGEDGYQMSAKIKKLGLGIPVIFLSARNDMDSKLQGLQIGEDYMVKPFDPRELLLRMRNMLEHHYGTFTQIKHLYIDAVTKKVFNESLHDEVLFTAIERKIF |

| | | | |
|---|---|---|---|
| | | | FYLYENRDSILTKEHFFEYLWQLEDRNPNIVNVHIKKIRAKINDQAGEMIENIYGEGYRLNTVVKK |
| D | *Bacillus subtilis* substr. subtilis str. 168 | *spo0A* | MEKIKVCVADDNRELVSLLSEYIEGQEDMEVIGVAYNGQECLSLFKEKDPDVLVLDIIMPHLDGLAVLERLRESDLKKQPNVIMLTAFGQEDVTKKAVDLGASYFILKPFDMENLVGHIRQVSGNASSVTHRAPSSQSSIIRSSQPEPKKKNLDASITSIIHEIGVPAHIKGYLYLREAISMVYNDIELLGSITKVLYPDIAKKFNTTASRVERAIRHAIEVAWSRGNIDSISSLFGYTVSMTKAKPTNSEFIAMVADKLRLEHKAS |
| D | *Bacillus subtilis* substr. subtilis str. 168 | *yrkQ* | MAYRILVVEDDEDIGDLLEESLTRAGYEVLRAKDGKRALQLVNDSLDLVILDIMMPGISGIETCQHIRKSSNVPILFLTARSSTLDKTEGLLAGGDDYMTKPFSEEELHARVIAQLRRYTIYQEKKEQEETFLIGGKLRVSEEFNEVWKEEKQIKLSDLEYRILKLLMNKRNKIFSAQNIYESVWGQPYFYCSNNTVMVHIRKLRSKIEDDPARPVYIKTEWGRGYRFGAS |
| D | *Bacillus subtilis* substr. subtilis str. 168 | *ytsB* | MFKLLLIEDDESLFHEIKDRLTGWSYDVYGIQDFSQVLQEFAAVNPDCVIIDVQLPKFDGFHWCRLIRSRSNVPILFLSSRDHPADMVMSMQLGADDFIQKPFHFDVLIAKIQAMFRRVHHYNTEPSTIKTWCGAAVDAEQNLVSNDKGSVELTKNEMFILKQLIEQKNKIVSREELIRSLWNDERFVSDNTLTVNVNRLRKKLDALQLGAYIETKVGQGYIAKEEDKFYD |
| D | *Bacillus subtilis* substr. subtilis str. 168 | *psdS* | MYRILLVEDDERIASLLGGHLQKYGYEVKIAEQLNDIKLEFAEMKPDLVLLDINLPFFDGFYWCRQIRTISNAPIIFISARTDELNQVMAIENGGDDYITKPFHLEVVMAKIKSVLRRTYGEYSPSLPQESRIVELGGLTIYPDQNEAEWNSVRILFSQKEFQLLSIFVREHKKIVSRDELLEALWDDVDFVDDNTLTVNVNRLRRKLENAGLTDCISTIRGQGYQFQVNRKDEAEC |
| D | *Bacillus subtilis* substr. subtilis str. 168 | *ywpD* | MKIRERFSMVDLPVLIITAAIIGHDKYKAFHAGANDILQKPYHYSEFMARIQNLIMMKHTANQATRMEMAFLQSQIKPHFLYNVLNTIISLTHLDIEKAREVTEEFTNYLRMSFDFQNTSAISSFRHELSIINSYLSIEKTRFSNRLEVLFDIDEDIDFILPPLMIQPLVENAVLHGVSKKRGGGWIKLTAKKQSKNEYHIKVEDNGPGITPEKQIDLLSTDFDRSVGLKNINQRLKHFCGSELMISSTPDAGTSVSMLIHLAETTGSPKELKDTERT |
| D | *Bacillus subtilis* substr. subtilis str. 168 | *tpeK* | MNKIMIVEDSEDIRGLLQNYLEKYGYQTVVAADFTAVLDVFLREKPDVVLLDINLPAYDGYYWCRQIRQHSTSPIIFISARSGEMDQVMAIENGGDDYIEKPFSYDIVLAKIKSQIRRAYGEYAAKQGEKVVEYAGVQLFVERFELRFQDEKSELSKKESKLLEVLLERGEKVTSRDRLMEKTWDTDIFIDDNTLNVYITRLRKKLRELNAPVSIEAVRGEGYQLRAQS |
| D | *Bacillus subtilis* substr. subtilis str. 168 | *ycbM* | MLVEDDHSISEMVDHYLTKEGFGIVHAFDGEEGIRLFQQGSYDLVLLDIMLPKLNGMDFLKIIREKSNIPVLMISAKDGDVDKALGLGFGADDYIAKPFSMIELTARVKAAIRRATQYSAEEPAVNKVIRIHQLAIDIDNVSVLKNGEPLQLTSTEWQLLCLFASNPKKVFTKEQIYRSVWNEEYFDDQNIINVHMRRLREKIEDDPSSPQYIKTLWGIGYKLGEF |
| D | *Bacillus subtilis* substr. subtilis str. 168 | *yvrH* | MENASILIVDDEKAIVDMIKRVLEKEGYRNILDAASAEEAIPVVKANKVDLIVLDVMMGGMSGFEACTLIREYSDAPIFFLTARSSDADKLSGFAVGADDYITKPFNPLELAARIRAHLKRTYQSKETSSNQTYTYDYFTFSPQNAELIVGGEAVACSA |

| | | | |
|---|---|---|---|
| | | | QLLQLLQYFCEHPNVVLSKDQIYEKVWGYPSYGDNNTVMVHIRKLREKIERDPSNPEYIVTVRGLGYRFIPNPEGKRS |
| D | *Pseudomonas aeruginosa* PAO1 | *PA5364* | MSKVSALVVDDAPFIRDLMKKGLRDNFPGLHIEEAVNGRKAQQLLSRQNVDLILCDWEMPEMSGLELLTWCRAQENLKTTPFIMVTSRGDKENVVQAIQAGVSDYIGKPFSNDQLVAKIKKALSRSGKLEALAAHAPRREIASGMANDSLAALTGGRAEVIKPAASPAKPAPAPKPASAPQASARPAGSGNPLGQAQLRLPQSSMPCVIKAVSLKEAQLVVKRADPLPQVLESAVLDLEENSDVARLNGYLHAIAALEPKPDSDWLLLTLRFVDRDPQKLDYLSRLIARGSTQKHYVPGA |
| D | *Pseudomonas aeruginosa* PAO1 | *PA2798* | MHKVSATLLIIDDDEVVRESLAAYLEDSNFKVLQALNGLQGLQIFESEQPDLVICDLRMPQIDGLELIRRIRQTASETPIIVLSGAGVMSDAVEALRLGAADYLIKPLEDLAVLEHSVRRALDRAYLRVENQRYRDKLEAANRELQASLNLLQEDQNAGRQVQMNMLPVTPWSIEGLEFSHRIIPSLYLSGDFVDYFRVDERRVAFYLADVSGHGASSAFVTVLLKFMTTRLLYESRRNGTLPEFKPSEVLAHINRGLINTKLGKHVTMLGGVIDLEKNSLTYSIGGHLPLPVLFVEGQAGYLEGRGLPVGLFDDATYDDRVMELPPSFSLSLFSDGILDVLPGATLKEKEASLPEQVAAAGGTLDGLRQVFGLANLAEMPDDIALLVLSRNLA |
| D | *Haemophilus influenzae* Rd KW20 | *HI0219a* | MEDVDLNIMVAKTILEKLGHHVDVATNGKQAITLFEKNVYDILLLDIKLPDMSGFEIAQYLRENYENGIYDFLPPMIAFTANVMQSEQEYLEMGMDGVLRKPISIKDLHHCLQQFFADESESIIEMNDDNELSEQFDLALIETLGKSQILENLSLFKQTMPNYLAQLSKDNMKETEDTAHKIKGAAASVGLNHLRQLADTLESAAKNSDVFNCGELIDKIGNLWLEDVEDLLKFCKF |
| D | *Salmonella enterica* subsp. Enterica serovar Typhimurium str. LT2 | *rssB* | MTQPLVGKQILIVEDEPVFRSLLDSWFSSLGATTALAGDGVDALELMGRFTPDLMICDIAMPRMNGLKLVENLRNRGDQTPILVISATENMADIAKALRLGVEDVLLKPVKDLNRLRETVFACLYPNMFNSRVEEEERLFRDWDAMVSNPTAAAQLLQELQPPVQQVISHCRINYRQLVSADQPGLVLDIAPLSDNELAFYCLDVTRAGDNGVLAALLLRALFNGLLQDQLGQQKHRLPELGALLKQVNHLLRQANLPGQFPLFVGYYHSELKNLILVSAGLNATLNTGAHQVQISSGVPLGTLGNAYLNQLSQRCDSWQCQIWGAGGRLRLMLSAE |

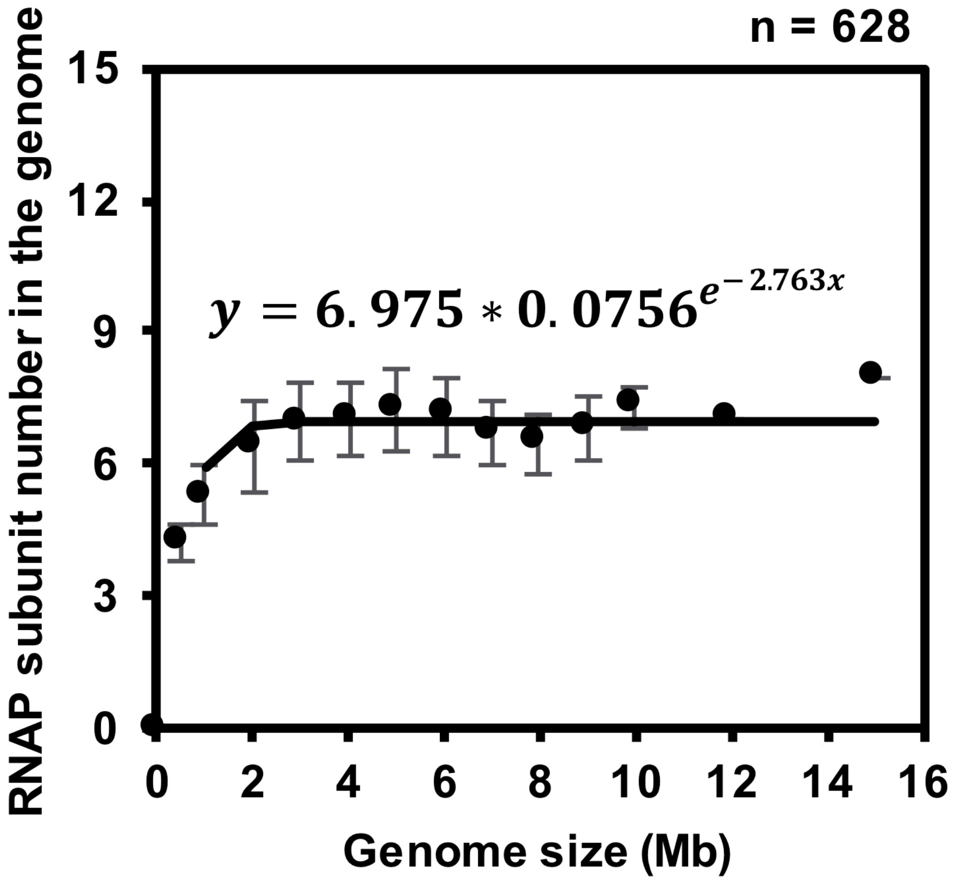


**Figure S1.** The correlation between genome size and the number of RNA polymerase subunit on bacterial genome. Eight groups are detected as RNA polymerase subunit from 628 species bacteria genomes in COG database (Tatusov et al., 2000) as the following; beta subunit as COG0085, beta' subunit as COG0086, sigma70/sigma32 subunit as COG0568, alpha subunit as COG0202, sigma24 subunit as COG1595, K/omega subunit as COG1758, sigma54 subunit as COG1508, and sigma subunit as COG1191. We used all of genome sequence of 628 species of bacteria registered in COG databank. The number of COGs involved in RNA polymerase subunit (y axis) was analyzed in comparison with genome size (x axis). The average of the number of COGs (black circle) and standard deviation (SD, error bar) was calculated for each genome size of 0.5, 1, 2, 3, 4, 5, 6, 7, 8, 9, 10, 12, and 15 Mb. The calculated data fitted Gompertz curve $y = ab^{e\wedge(-cx)}$ with y is the number of COGs and x is the genome size. The formula of fitted Gompertz curve are shown in the graph.

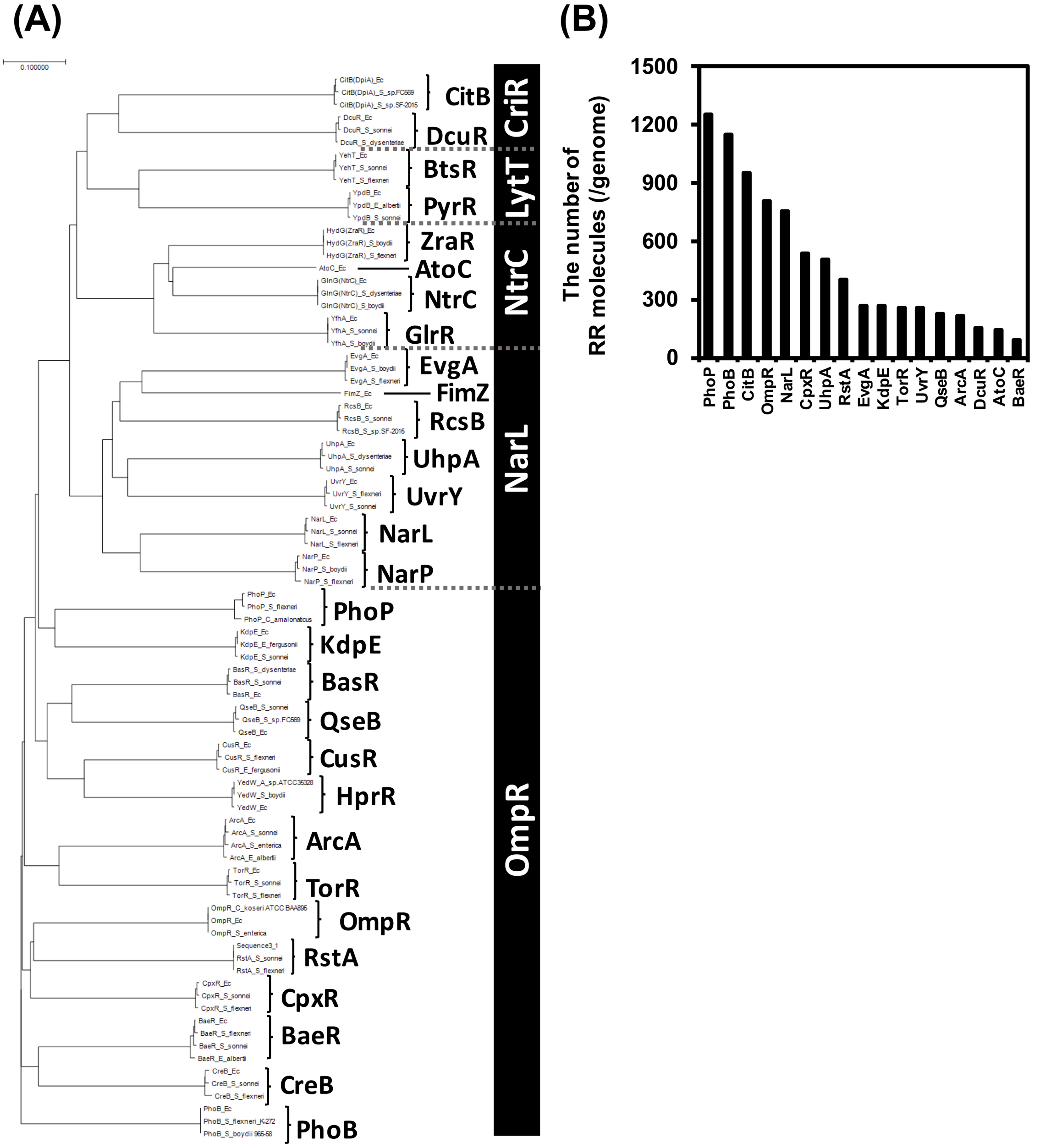


**Figure S2.** Classification of two-component system RRs in *E. coli*. [A] The dendrogram of response regulators of *E. coli*. Cluster analysis was performed for RRs of *E. coli* and two of related species (*E. albertii, S. enterica, S. sonnei, S. flexneri* K-272, *S. dysenteriae, S. boydii* 965-58, *C. freundii, E. fergusonii,* and *C. koseri* ATCC BAA895) with ClustalW software, resulting in each of RR family shown in the right side. [B] The amount of intracellular RR molecules in *E. coli* K-12 cell. The intracellular levels of 65 species of transcription factor with known function in *E. coli* K-12 W3110 at various phases of cell growth has showed the order of intracellular response regulators. (Ishihama et al., 2014). Y axis shows the number of RR molecules per genome as arranged from Ishihama et al. (2014).

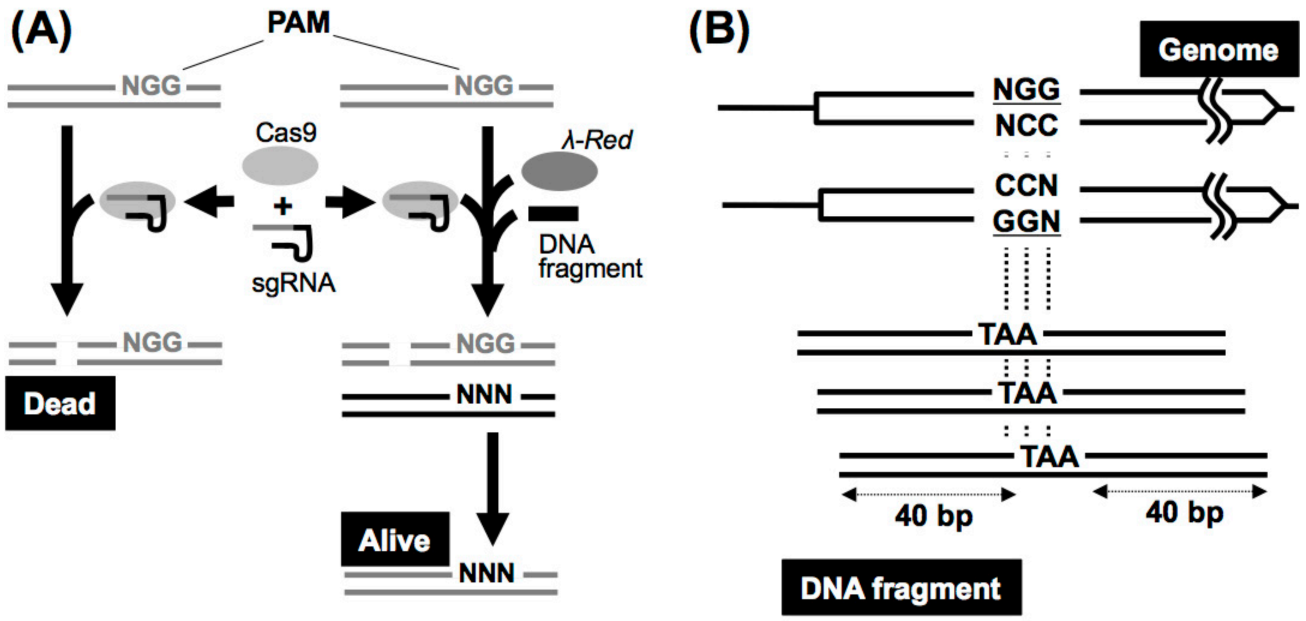


**Figure S3.** Homologous sequence integration (HoSeI) method for multi-gene knockout on *E. coli* genome. [A] The overview of HoSeI system. HoSeI system is based on previously reported genome editing method using CRISPR-Cas (Jiang et al., 2014). The sgRNA expressed from psgRNA and *Streptococcus pyogenes* Cas9 endonuclease expressed from pCas cause site-specific double-strand break at the recognized site by protospacer adjacent motif (PAM) for sgRNA, resulting in cell death of *E. coli* (left). In HoSeI system, the recombination of DNA fragment by lambda-Red recombinase recovers the digestion of *E. coli* genome and enables *E. coli* cell to avoid cell death (right). [B] The design of DNA fragment for replacing PAM with nonsense codon. To knockout protein-coding gene, we designed the DNA fragment containing TAA, introducing both nonsense codon and mutated PAM, with 40 bp-long homology arms on both sides.

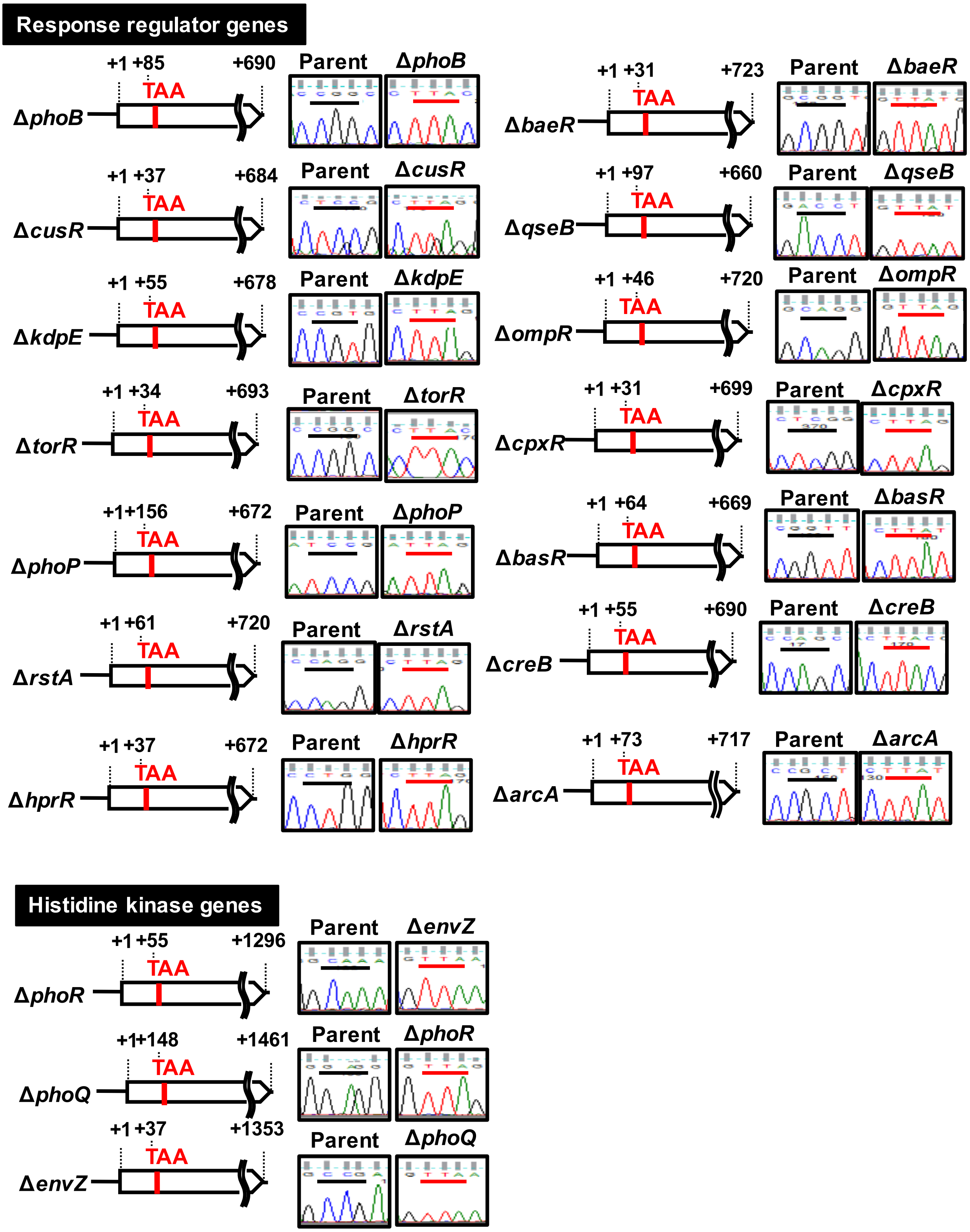


**Figure S4.** Construction of RR or SK-coding gene knockout on *E. coli* genome. We isolated RR or their cognate SK gene knockout strains using HoSeI method (Fig. S3). The introduction of stop codon on target gene was confirmed by DNA sequencing on the amplified DNA.

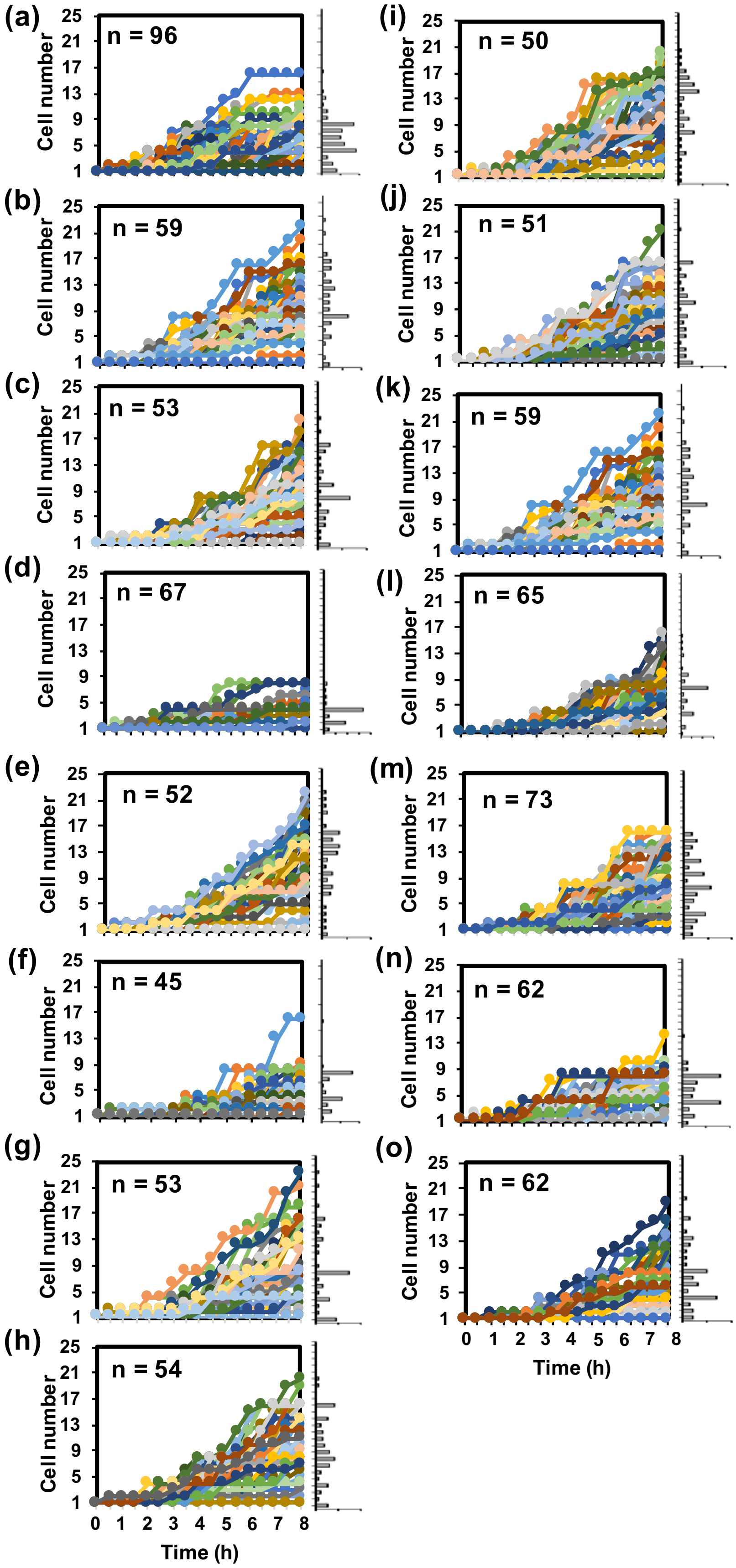


**Figure S5.** Single cell analysis of adaptive growth of single RR gene knockout *E. coli* strain. Strains were

inoculated in M9 glucose medium and shaken overnight at 37$^{o}$C. The cultures were washed with M9 medium three times and diluted 10-fold into the medium. The diluted cultures were spread on M9 glucose agar plate. The piece of agar from the plates were cut and put on a slide glass. The preparation was sealed on a glass coverslip with nail polish. The cells were imaged on a microscope (IX81, Olympus) using 100x/NA 1.4 objective lens (M Plan Apochromat MPLAPON-Oil, Olympus) and Retiga EXi Fast1394 CCD camera (Q Imaging) every 30 min until 8 hours. Temperature was maintained at 30$^{o}$C using a closed circulation system (EYELA). Image acquisition and microscope control were performed with Image Pro Plus (Nippon roper). The cell division of each *E. coli* cell (hours, showed in x axis) and the population of cell in each microcolony (y axis) were measured by ImageJ. The histograms of cell population in a microcolony at after 8 hrs were showed on the right side of each graph. Each graph shows the parent strain W3110 type A (a), Δ*phoB* (b), Δ*cusR* (c), Δ*kdpE* (d), Δ*torR* (e), Δ*phoP* (f), Δ*rstA* (g), Δ*hprR* (h), Δ*baeR* (i), Δ*qseB* (j), Δ*ompR* (k), Δ*cpxR* (l), Δ*basR* (m), Δ*creB* (n), and Δ*arcA* (o). The number of measured cell (n) are shown in each graph.

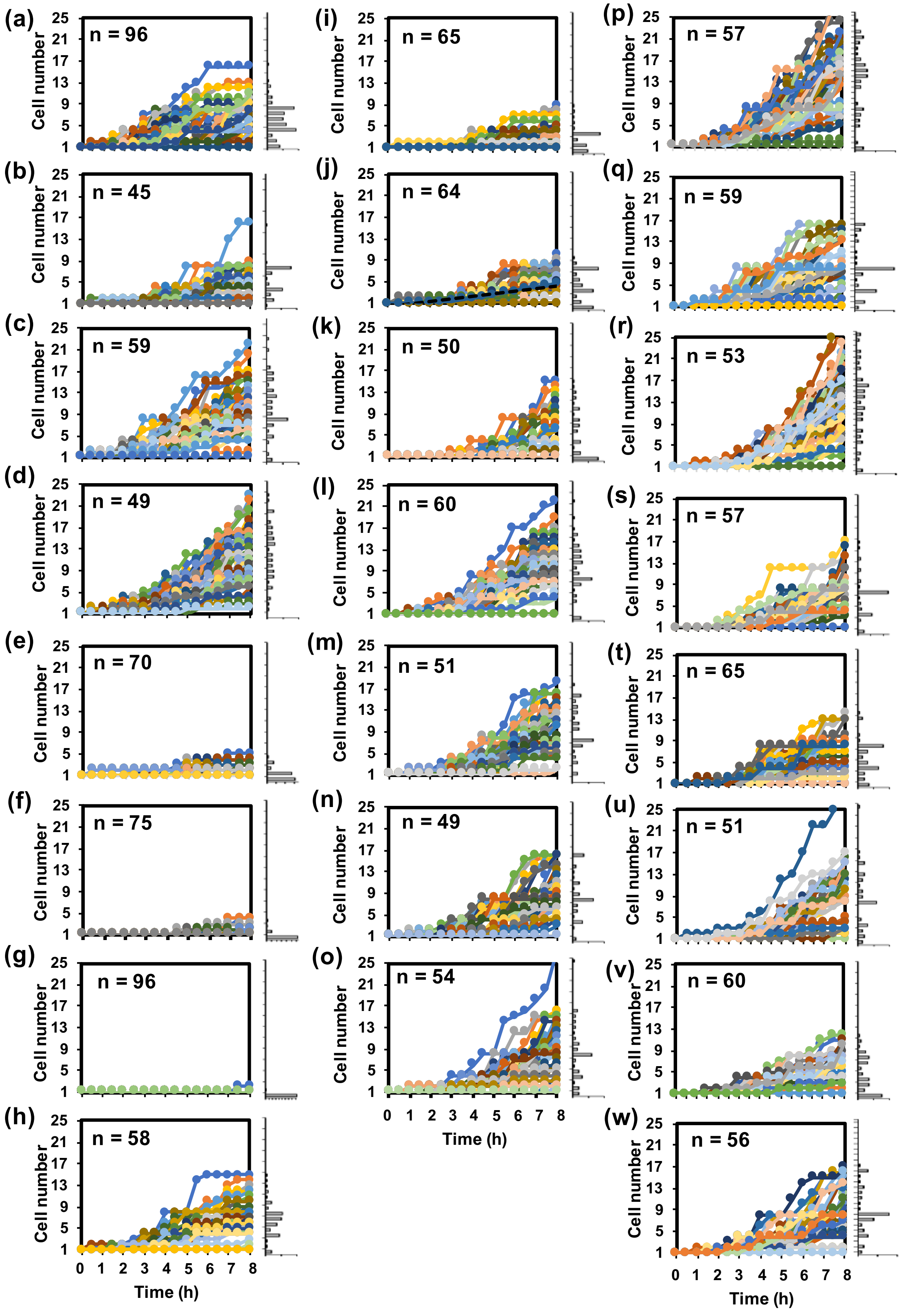


**Figure S6.** Single cell analysis of adaptive growth of multi-gene knockout *E. coli* strains. Strains were

cultured, spread on M9 glucose agar plate, and imaged on a microscope as described in Fig. S5. The cell division (hours, showed in x axis) and the population of cell in each microcolony (y axis) were measured and the histograms of cell population in a microcolony at after 8 hrs were showed on the right side of each graph. Each graph shows the parent strain W3110 type A (a), Δ*phoP* (b), Δ*phoB* (c), Δ*ompR* (d), Δ*phoPphoB* (e), Δ*phoP*Δ*ompR* (f), Δ*phoB*Δ*ompR* (g), Δ*phoP*Δ*phoB*Δ*ompR* (h), Δ*phoQ* (i), Δ*phoR* (j), Δ*envZ* (k), Δ*phoQ*Δ*phoR* (l), Δ*phoQ*Δ*envZ* (m), Δ*phoR*Δ*envZ* (n), Δ*phoQ*Δ*phoR*Δ*envZ* (o), Δ*phoP*Δ*phoQ* (p), Δ*phoB*Δ*phoR* (q), Δ*ompR*Δ*envZ* (r), Δ*phoP*Δ*kdpE* (s), Δ*phoB*Δ*creB* (t), Δ*ompR*Δ*cpxR* (u), Δ*ompR*Δ*rstA* (v), and Δ*rstA*Δ*cusR*Δ*hprR* (w). The number of measured cell (n) are shown in each graph.

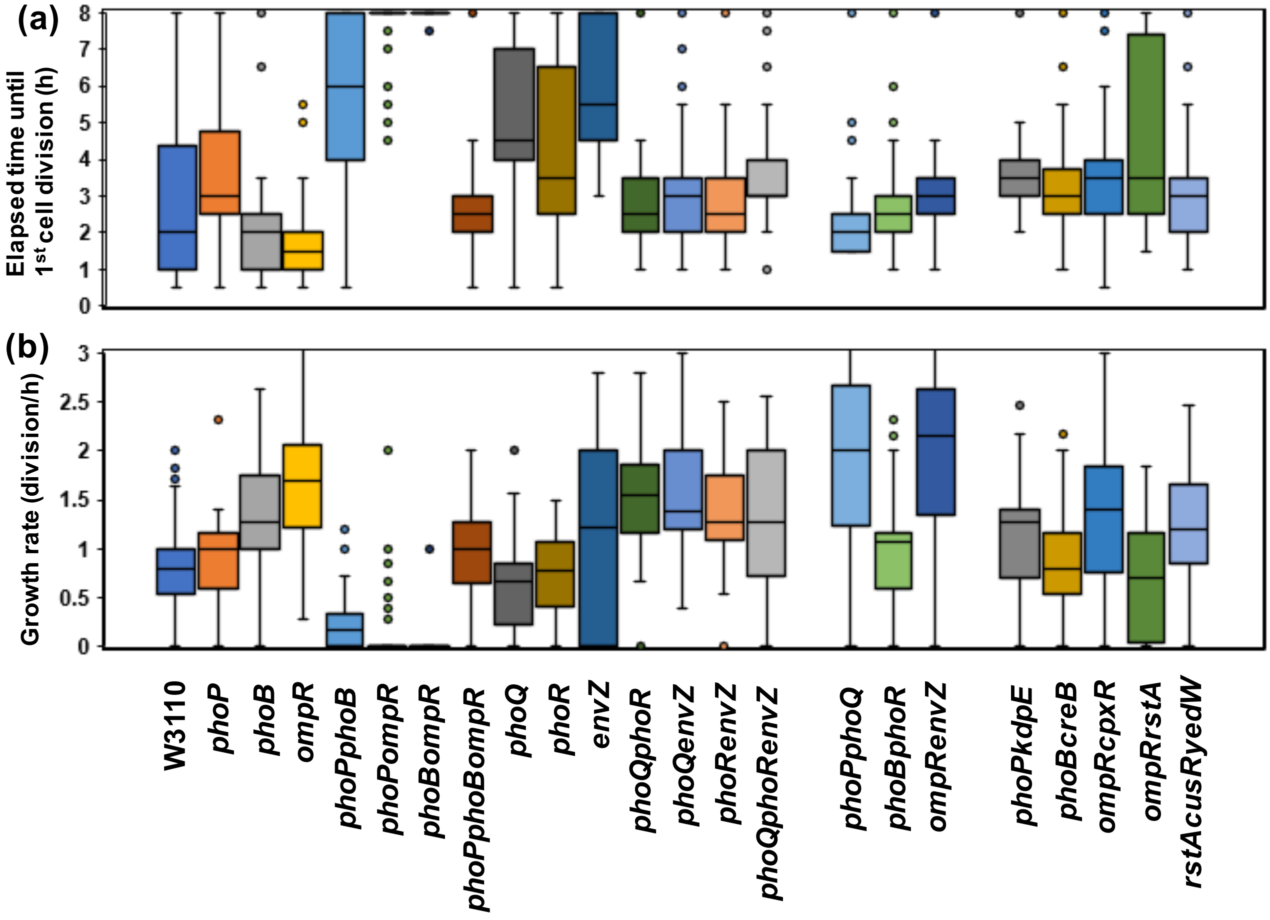


**Figure S7.** The distribution of cell growth values of *E. coli* strains. Based on the measured data (Figs. S6), the elapsed time until cell division (a) and growth rate of single *E. coli* cell (b) were calculated and shown as box plots with error bars and outliers.

**(a) Elapsed time until 1st cell division**

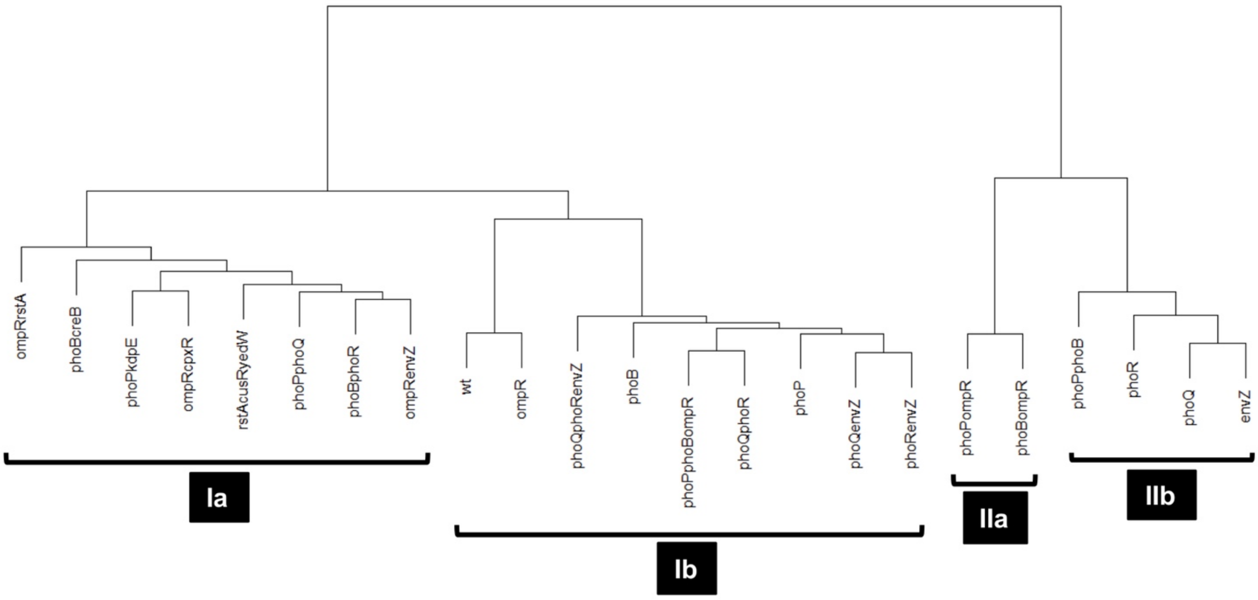


**(b) Growth rate**

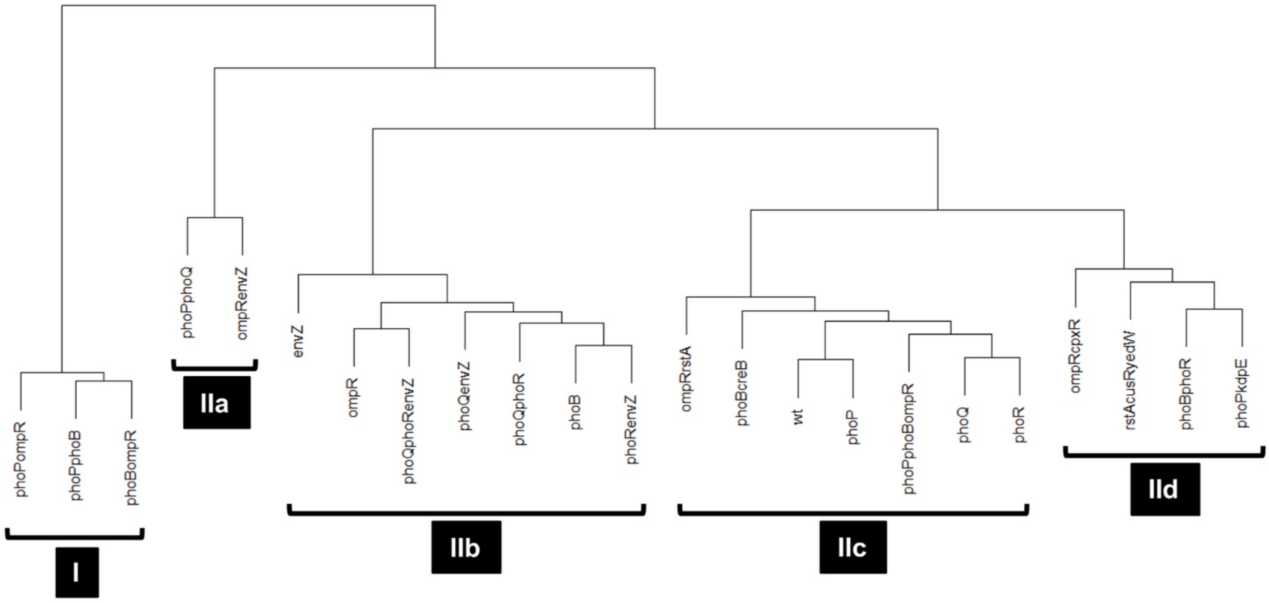


**Figure S8.** Cluster analysis using values for *E. coli* cell growth of strains. Cluster analysis was performed with R software. The presenting data of the elapsed time (a) and the growth rate (b) were prepared for Ward's method. Hierarchical clustering dendrogram was calculated using Euclidean distances.

**Reference**


Burton NA. *et al*. Novel aspects of the acid response network of *E. coli* K-12 are revealed by a study of transcriptional dynamics. *J Mol Biol*. 401, 726-42, DOI: https://doi.org/10.1016/j.jmb.2010.06.054. (2010)

Guzman LM. *et al*. Tight regulation, modulation, and high-level expression by vectors containing the arabinose PBAD promoter. *J Bacteriol*. 1995 177, 4121-30, DOI: https://doi.org/10.1128/jb.177.14.4121-4130 (1995).

Ishihama, A. *et al.* Intracellular concentrations of 65 species of transcription factors with known regulatory functions in *Escherichia coli. J. Bacteriol.* 196, 2718–2727, DOI: https://doi.org/10.1128/JB.01579-14 (2014).

Jiang, Y. *et al.* Multigene editing in the *Escherichia coli* genome via the CRISPR-Cas9 system. *Appl. Environ. Microbiol.* 81, 2506–2514, DOI: https://doi.org/10.1128/AEM.04023-14 (2015).

Jishage, M. & Ishihama, A. Variation in RNA polymerase sigma subunit composition within different stocks of *Escherichia coli* w3110. *J. Bacteriol.* 179, 959–963, DOI: https://doi.org/10.1128/jb.179.3.959-963.1997 (1997).

Tatusov, R. L., Galperin, M. Y., Natale, D. A. & Koonin, E. V. The COG database: A tool for genome-scale analysis of protein functions and evolution. *Nucleic Acids Res.* 28, 33–36, DOI: https://doi.org/10.1093/nar/28.1.33 (2000).
